# Parametric and Nonparametric Statistical Methods for Genomic Selection of Traits with Additive and Epistatic Genetic Architectures

**DOI:** 10.1534/g3.114.010298

**Published:** 2014-04-09

**Authors:** Réka Howard, Alicia L. Carriquiry, William D. Beavis

**Affiliations:** * Department of Statistics, Iowa State University, Ames, Iowa 50011; † Department of Agronomy, Iowa State University, Ames, Iowa 50011

**Keywords:** parametric, nonparametric, genomic selection, epistasis, prediction, GenPred, Shared data resources

## Abstract

Parametric and nonparametric methods have been developed for purposes of predicting phenotypes. These methods are based on retrospective analyses of empirical data consisting of genotypic and phenotypic scores. Recent reports have indicated that parametric methods are unable to predict phenotypes of traits with known epistatic genetic architectures. Herein, we review parametric methods including least squares regression, ridge regression, Bayesian ridge regression, least absolute shrinkage and selection operator (LASSO), Bayesian LASSO, best linear unbiased prediction (BLUP), Bayes A, Bayes B, Bayes C, and Bayes C*π*. We also review nonparametric methods including Nadaraya-Watson estimator, reproducing kernel Hilbert space, support vector machine regression, and neural networks. We assess the relative merits of these 14 methods in terms of accuracy and mean squared error (MSE) using simulated genetic architectures consisting of completely additive or two-way epistatic interactions in an *F*_2_ population derived from crosses of inbred lines. Each simulated genetic architecture explained either 30% or 70% of the phenotypic variability. The greatest impact on estimates of accuracy and MSE was due to genetic architecture. Parametric methods were unable to predict phenotypic values when the underlying genetic architecture was based entirely on epistasis. Parametric methods were slightly better than nonparametric methods for additive genetic architectures. Distinctions among parametric methods for additive genetic architectures were incremental. Heritability, *i.e.*, proportion of phenotypic variability, had the second greatest impact on estimates of accuracy and MSE.

Complex quantitative traits are measured on a continuous scale and are controlled by a network of many genes, by the environment, and by genetic by environment interactions. Most traits of economical interest in agriculture (*e.g.*, grain yield) are measured on continuous scales, *i.e.*, they are quantitative. Understanding the complexity of these traits and accounting for the effects that are contributed by these genes and their interactions is not trivial.

The gene-by-gene interaction or epistasis is an important research topic in quantitative genetics. Epistasis can be modeled in different ways (Cordell 2002). Physiological epistasis is the difference in the phenotype when the genotype at a locus is influenced by the genotype at another locus or loci. Fisher (1918) defined epistasis as the deviation of the genotypic value from the contribution of the sum of additive effects at all functional loci in the genome. Fisher’s definition of epistasis is also known as statistical epistasis and has been used to quantify deviations from independence (Wilson 2004). Epistasis has an important role in accounting for the genetic variation for quantitative traits, and excluding it from the prediction equations for simplicity can result in poor predictions of genetic gain (Cooper *et al.* 2002).

Most simulation studies of genomic selection (GS) methods (Meuwissen *et al.* 2001) have considered genetic architectures in which the number and relative magnitudes of quantitative trait loci (QTL) have varied. To our knowledge, no studies of GS methods have considered epistatic genetic architectures, although Gianola *et al.* (2006) predicted nonparametric methods would be better-suited for epistatic genetic architectures. Although theoretic models predict a significant role for epistasis in speciation (Dobzhansky 1937; Mayr 1942), adaptation (Lewontin 1974; Wade 2000), and canalization (Waddington 1949; Rice 1998), there is little empirical evidence from biometric studies of significant epistatic contributions to genetic variability. Biometric approaches, however, average across epistatic genotypic values at individual loci and contribute primarily to additive genetic variance (Cockerham 1954; Cheverud and Routman 1995). With development of low-cost high-throughput marker technologies, it has become possible to estimate epistatic interactions based on genotypic values for all possible pairwise genotypes in genome-wide association studies, although searches for higher-order interactions are still limited by experimental and computational resources (Moore and Williams 2009). These studies are beginning to reveal that epistasis is not the exception, but rather the most prevalent form of genetic architecture for quantitative traits (Flint and Mackay 2009; Huang *et al.* 2012). Nonetheless, it was hypothesized that GS should provide accurate predictions because epistatic gene action will be translated primarily into additive genetic variance (Crow 2010). Thus, for purposes of this study, we decided to evaluate GS methods for an extreme case of epistasis with 10 pairs of loci, each consisting of two alleles at equal frequencies and modeled using the principle of orthogonality (Goodnight 2000).

The development of DNA markers in the 1980s was an important step in the process of identifying DNA segments that are statistically associated with quantitative traits, *i.e.*, QTL mapping and for marker-assisted selection (MAS). In MAS, markers and phenotypic information are used to guide indirect selection of a trait of interest (Fernando and Grossman 1989). This approach is considered an improved and more efficient method for selection in plant breeding relative to phenotype pedigree–based approaches (Mohan *et al.* 1997). Extensive resources have been devoted to develop QTL mapping methodology as a component of MAS (Young 1996; Melchinger *et al.* 1998). Marker-assisted backcrossing (MABC) is one of the simplest examples of MAS. In MABC, genomic regions defined by markers closely linked to QTL are identified. These genomic regions are then introgressed into the elite lines through backcrossing (Bernardo 2010). In MABC, a plant with a desired gene, called a donor parent, is crossed with an elite or breeding line, called a recurrent parent. The goal is to introgress the desired gene into the genome of the recurrent parent (Visscher *et al.* 1996). Developing varieties can also involve accumulating multiple desired genes into a recurrent parent. The marker-assisted process for alleles at multiple loci is called gene pyramiding. MAS is widely used in gene pyramiding because the use of molecular markers gives the advantage of selecting the desired plants without extensive phenotyping. With traditional phenotyping, it is often impossible to distinguish among plants with all desirable alleles and the plants with some of the desirable alleles (Huang *et al.* 1997).

MAS has been shown to be efficient and effective for traits that are associated with one or a few major genes with large effect but does not perform as well when it is used for selection of polygenic traits (Bernardo 2008). QTL detection also results in some false-negative and false-positive rates, and further QTL mapping does not guarantee that estimates of genetic effects are correct (Beavis 1994). Also, for MAS to be useful, the interaction between the QTL and the genetic background has to be minimal, so the QTL has the same effect in different genetic backgrounds (Bernardo 2010 p. 223). The genetic background of an organism refers to all of its alleles at all loci that can interact with the locus where the QTL is located (Yoshiki and Moriwaki 2006).

The parametric models and statistical methods introduced for QTL mapping and MAS do not address genetic improvement for quantitative traits that are influenced by a large number of genes with small effects. Some of the statistical challenges arising in MAS include the specification of threshold for multiple testing, the “large p, small n” problem [which refers to the situation when the number of predictors, p (marker data points) greatly exceeds the number of individuals, n, that have been evaluated in the study], difficulty of interpretation of effects due to collinearity among the explanatory/predictor variables, model assumptions that cannot be satisfied, and nonadditivity among genetic effects.

With advanced molecular techniques that provide dense marker maps, it is possible to overcome some shortcomings of MAS. Meuwissen *et al.* (2001) proposed predicting the genotypic value for individuals using all marker information simultaneously. Their proposed method and the subsequent derivative methods have been referred to as GS. They modeled the associations between the markers and a phenotype focusing on calculating a breeding value for an individual (which can be calculated as the sum of the average effect of the alleles for the individual’s genotype) instead of identifying significant marker–trait associations. In their approach they estimated the effect of each QTL and then used the sum of all estimates to calculate a genotypic value for the individual.

In GS, individuals with both phenotypic and marker information (called the training set) are used to model the association between the phenotype and the genotype. The model is used to predict the phenotypic value of individuals for which only the marker information is available (called the validation set or testing set). In GS, all available markers are included in the model, not just those above a significant threshold, thus eliminating the problem of multiple testing.

Effort is underway to find ways to model epistasis, the gene-by-gene interaction. In the presence of epistasis, the effect of one locus changes the effect of another locus on the phenotype. Usually several loci are involved, which means that multiway interactions may need to be modeled. Because the volume of marker data points available is huge, the number of epistatic interactions can be overwhelming and computationally intractable to estimate with parametric methods (Moore and Williams 2009).

More recently, Gianola *et al.* (2006) stated that parametric approaches to GS have several drawbacks. The parametric model assumptions do not always hold (*e.g.*, normality, linearity, independent explanatory variables), which suggests the use of nonparametric methods. Also, the convenient partitioning of genetic variance into additive, dominance, additive × additive, additive × dominance, etc., only holds under conditions of linkage equilibrium, random mating of male and female parents, no inbreeding, no assortative mating, no (natural or artificial) selection, and no genotyping errors. In breeding programs, these conditions are all violated. Gianola *et al.* (2006) proposed nonparametric and semi-parametric methods to model the relationship between the phenotype and the markers that are available within the GS framework. Gianola *et al.* (2006) proposed nonparametric methods capable of accounting for complex epistatic models without explicitly modeling them.

Herein, we review some existing statistical methods used in GS. First, we discuss the parametric methods in more detail. Then, we focus on the nonparametric and semi-parametric methods. Among parametric methods, we review linear least squares regression, penalized ridge regression, Bayes ridge regression, least absolute shrinkage and selection operator (LASSO), and Bayes LASSO methods, best linear unbiased prediction (BLUP), and some Bayesian alternatives used in GS (Bayes A, Bayes B, Bayes C, and Bayes C*π*). We also explain the nonparametric kernel regression using the Nadaraya-Watson estimator (NWE) and the semi-parametric reproducing kernel Hilbert space (RKHS) regression. Finally, we describe support vector machine (SVM) regression and neural networks (NN) applications to GS. de los Campos *et al.* (2013) give an overview of some of the parametric methods used in GS, and Gianola *et al.* (2010) provide information about some of the nonparametric models used in GS. Heslot *et al.* (2012) compared some parametric and nonparametric GS methods. However, they did not consider epistatic genetic architectures in their simulated data. Daetwyler *et al.* (2010) discussed the impact of the genetic architecture in GS, but they defined genetic architecture by the effective population size and the number of QTL.

Here, we use simulated data to compare the performance of the parametric models with the nonparametric procedures for predicting the genetic value for individuals in a *F*_2_ and a backcross (BC) populations. We simulate the *F*_2_ and the BC populations with low and high heritabilities and compare the two extreme genetic architectures. One architecture had only additive genetic effects from alleles at 30 loci, and the other had only two-way epistatic genetic effects among 30 loci. The performance of the methods is illustrated by comparing the accuracy of prediction, which we defined by the correlation between the true phenotypic value and the predicted phenotypic value and the mean squared error (MSE). We demonstrate the advantage of some nonparametric methods for the epistatic genetic architecture. Because the results for the *F*_2_ and BC populations were similar, we only illustrate the *F*_2_ population in this article. In the Supporting Information, we provide accuracy and MSE values for a simulated BC population with low and high heritabilities and with two extreme genetic architectures.

## Parametric methods in genome-wide selection

### Linear least-squares regression model:

In GS, the main goal is to predict the individual’s breeding value by modeling the relationship between the individual’s genotype and phenotype. One of the simplest models is:

$$y_{i}=\mu +\sum_{j=1}^{p}X_{ij}m_{j}+e_{i},$$ (1)

where *i* = 1…*n* individual, *j* = 1…*p* marker position/segment, *y_i_* is the phenotypic value for individual *i*, *μ* is the overall mean, *X_ij_* is an element of the incidence matrix corresponding to marker *j*, individual *i*, *m_j_* is a random effect associated with marker *j*, and *e_i_* is a random residual. Typically, the residual term, *e*, is chosen to have a normal distribution with mean of 0 and variance of $\sigma_{e}^{2}$. The model for the data vector **y** can be written as:

$$y_{n\times 1}=\mu_{n\times 1}+X_{n\times p}m_{p\times 1}+e_{n\times 1}.$$ (2)

To estimate (*μ*, *m*), we can use least squares to minimize the sum of squared vertical distance between the observed response and the estimated response, which can be represented as |**y** − **Xm**|^2^ (where | denotes the norm of a vector). The estimate of **m** obtained by solving the linear equations *X*′*X***m** = *X*′**y**. Then, it is estimated as $\hat{m}={\left(X^{\prime }X\right)}^{-1}X^{\prime }y$. For more details about linear models, the reader can refer to *Linear Models in Statistics* (Schaalje and Rencher 2000) or *Linear Models with R* (Faraway 2006). The elements of the design matrix **X** depend on the number of different alleles present. For example, individuals having marker genotypes *AA*, *Aa*, *aa* have elements coded as −1, 0, and 1 in *X_ij_*, respectively.

One obvious problem with linear regression is that usually the number of markers (explanatory variables) available is much greater than the number of individuals with phenotypic information (response variables), which means that *p* is much greater than *n*, and it is impossible to perform the estimation. Using a subset of the markers can be an alternative (using a variable selection method like the forward, backward, or stepwise selection procedure) (George 2000), but it can still perform poorly if the relative ratio of the number of markers and the number of individuals is large or has multicollinearity, *e.g.*, linkage disequilibrium (LD) exists among the markers.

Meuwissen *et al.* (2001) used a modification of least squares regression for GS. First, they performed least squares regression analysis on each segment separately using the model **y** = *μ* + **X_j_***m_j_* + **e**, where **y** is the vector of the phenotypic information, *μ* is the overall mean vector, *X_j_* is the *j^th^* column of the design matrix corresponding to the *j^th^* segment, *m_j_* is the genetic effect associated with the *j^th^* segment, and **e** is the vector of the error terms. By plotting the log likelihood of this model, segments with significant effects were found. The segments with significant effect (QTL) were used for simultaneous estimation by the model: $y=\mu +\sum_{j=1}^{q}X_{j}m_{j}+e$, where *q* is the number of QTL. With this approach, they eliminated the problem of having more predictor (explanatory/independent) variables than regressands (response/dependent variables), but it does not fully take advantage of the whole marker information because only markers with a significant effect are included in the final model. To overcome some of the drawbacks of the linear regression approach, other methods for GS have been introduced.

### Ridge regression:

In marker data, it is very likely that multicollinearity exists. As discussed in the previous section, multicollinearity can negatively affect the performance of variable selection methods. Further, least squares equations are inefficient when the determinant of the matrix **X**′**X** is close to zero due to column dependencies. Using a penalized regression model (ridge regression of Hoerl and Kennard 1970a,b) can be a solution to this problem. The goal is to derive an estimator of **m** with smaller variance than the least squares estimator. There is a “price to pay” in that the ridge regression estimator of **m** is biased; the increase in bias is more than compensated by the decrease in variance, which results in an estimator ${\hat{m}}_{R}$ with smallest MSE. Another advantage of ridge regression is that it can be used when a large amount of marker information is available, so it can overcome the $“p>n^{\prime \prime }$ problem.

Ridge regression adds an extra term to the likelihood function to shrink the regression coefficients by an amount depending on the variance of the covariates. It removes the problem of the columns of the design matrix being dependent on each other and, hence, the **X**′**X** matrix will be nonsingular. Instead of minimizing the sum of squared residuals, ridge regression minimizes the penalized sum of squares |**y** − **Xm**|^2^ + *λ*^2^**m**′**m**, where *λ* is the penalty parameter, and the estimate of the regression coefficient is given by: $\hat{m}={\left(X^{\prime }X+\lambda I\right)}^{-1}X^{\prime }y$, where **I** is a *p* × *p* identity matrix. The penalty parameter *λ* can be calculated by several different methods, for example, by plotting $\hat{m}$ as a function of *λ* and choosing the smallest *λ* that results in a stable estimate of $\hat{m}$. Another way to choose *λ* is by an automated procedure proposed by Hoerl *et al.* (1975). They claimed that a reasonable choice of *λ* is given by: $\lambda =\frac{rs^{2}}{\left(\hat{m}\right)\prime \left(\hat{m}\right)}$, where *r* is the number of parameters in the model not counting the intercept, *s*^2^ is the residual mean square obtained by linear least squares estimation, and $\hat{m}$ is the vector of least squares estimates of regression coefficients.

Meuwissen *et al.* (2001) implemented ridge regression in GS by assuming that the marker effects (*m_j_*′s *j* = 1…*p*) were random, and they were drawn from a normal distribution with $Var\left(m_{j}\right)=\sigma_{m}^{2}$, where $\sigma_{m}^{2}=\sigma_{a}^{2}/n_{k}$ $\sigma_{a}^{2}$ represents additive genetic variance expressed among individuals and *n_k_* is the number of marker loci (Habier *et al.* 2007).

It can be shown that ridge regression is a special case of the BLUP (Ruppert *et al.* 2003), which we demonstrate after introducing BLUP. Thus, the mixed linear model can be implemented. Within the mixed model context, the restricted maximum likelihood (REML) estimation is a good choice for finding a reasonable value for the penalty parameter and estimating the variance components (Henderson 1988). Piepho (2009) discusses some models that feature the ridge regression in terms of mixed models and uses REML for variance and penalty parameter estimation in GS.

Ridge regression can also be viewed from a Bayesian perspective. In this case, we assume that the parameter vector **m** is random. We can account for the belief that the estimator of **m** has a small variance by a choice of a prior distribution. In particular, we can suppose that **m** ∼ *N*(0, ∑*_β_*), where ∑*_β_* is a known covariance matrix (de Boer *et al.* 2005). Given that the likelihood of *y_i_* (*i* = 1, 2, …*n*, where *n* is the number of individuals) has a normal distribution with mean $\sum_{j=1}^{p}x_{ij}m_{j}$ and variance *σ*^2^, the Bayesian estimator of **m** is the mean of the posterior distribution, and it is given by ${\hat{m}}_{BRR}={\left(\sigma^{2}\sum_{\beta }^{-1}+X\prime X\right)}^{-1}X^{\prime }y$ (Judge *et al.* 1985 p. 286). Comparing ${\hat{m}}_{BRR}$ to ${\hat{m}}_{RR}$, we can see that they are identical if $\sum_{\beta }^{-1}=\frac{\lambda }{\sigma^{2}}I$.

Pérez *et al.* (2010) discussed the application of Bayesian ridge regression in GS. They assumed that the marker effects are independent and identically distributed (iid) and have a normal prior distribution with mean 0 and variance $\sigma_{\beta }^{2}$, where: $p\left(m|\sigma_{\beta }^{2}\right)=\prod_{i=1}^{n}N\left(m_{j}|0,\sigma_{\beta }^{2}\right)$. Then the mean of the posterior distribution ${\hat{m}}_{BRR}$ is equivalent to ${\hat{m}}_{RR}$ if $\lambda =\frac{\sigma_{\beta }^{-2}}{\sigma^{2}}$.

### Best linear unbiased prediction:

The BLUP theory and the mixed model formulation were first discussed by Henderson (1949), and they were influential for selection purposes in animal breeding (Henderson 1959). BLUP is a statistical procedure, and it is useful in situations when the data available are unbalanced (for example, in different locations the number of individuals is not the same), and it can accommodate family information (Bernardo 2010). Since Henderson’s first work in BLUP, the theory has been widely expanded (Henderson 1959, 1963, 1975a, 1975b; Harville 1976). Since the 1990s, BLUP has been used not only in animal breeding applications (Henderson 1984) but also in plant breeding (Bernardo 1994).

BLUP was proposed as a tool in GS by Meuwissen *et al.* (2001). The random effects model can be written in the form:

$$y=\mu +\sum_{j=1}^{p}Z_{j}m_{j}+e,$$ (3)

where **y** is the (*n* × 1) phenotypic data vector, *μ* is the (*n* × 1) overall mean vector, **Z_j_** is the *j^th^* column of the design matrix, *m_j_* is the genetic effect associated with the *j^th^* marker, and *p* is the number of markers. The intercept, *μ*, is fixed, and *m_j_* is the random effects with *E*(*m_j_*) = 0, $Var\left(m_{j}\right)=\sigma_{m_{j}}^{2}$, *Var*(**e**) = *σ*^2^**I**, and *Cov*(**m**, **e**) = 0. In the statistical literature, the vector of random effects is usually denoted by **u** instead of **m**. If other covariates are available, then we replace the intercept *μ* by **X***β* to include all the fixed effects. Then, we can write:

y=Xβ+Zm+e, (4)

where *β* is a *p*_1_ × 1 vector of unknown fixed effects where usually the first element is the population mean, and **X** is the incidence matrix that relates **y** to *β*. The above equation is generally called a mixed model (or mixed effects model). The vector *β* is estimated by the best linear unbiased estimator (BLUE). In the biological literature, the term BLUP is occasionally used loosely and refers to both BLUE and BLUP. BLUP is the predictor of the random effects. It is a linear function of the data vector **y**. Within the linear functions of the data it is unbiased, which means that the expected value of the prediction is the same as the population parameter, and it can be formulated as $E\left(\hat{m}\right)=E\left(m\right)$. In addition within the unbiased linear predictors, it is the best in the sense of minimizing the MSE. BLUE is similar to BLUP in that it is a linear function of the data **y**, it is unbiased among the linear estimators, and it is best in the sense that it minimizes the MSE.

Henderson (1953) proposed that the BLUE and BLUP of (*β*, **m**) be obtained by maximizing the joint likelihood of (**y**, **m**) given by:

$$\begin{array}{l} L\left(y,m\right)=f\left(y|m\right)f\left(m\right)\\ =\frac{1}{{\left(2\pi \right)}^{n/2}{\left|R\right|}^{1/2}}\left[-\frac{1}{2}\left(y-X\beta -Zm\right)\prime R^{-1}\left(y-X\beta -Zm\right)\right]\times \frac{1}{{\left(2\pi \right)}^{p/2}{\left|G\right|}^{1/2}}\left[-\frac{1}{2}m\prime G^{-1}m\right]. \end{array}$$

By maximizing the likelihood *L*(**y**, **m**) with respect to *β*, **m** and equating it to zero, we obtain a set of linear equations [known as Henderson’s mixed model equations (MME)]:

$$\left(\begin{array}{cc} X\prime R^{-1}X & X\prime R^{-1}Z\\ Z\prime R^{-1}X & Z\prime R^{-1}Z+G^{-1} \end{array}\right)\left(\begin{array}{c} \hat{\beta }\\ \hat{m} \end{array}\right)=\left(\begin{array}{c} X\prime R^{-1}y\\ Z\prime R^{-1}y \end{array}\right),$$

where *R* = *Var*(**e**) and *G* = *Var*(**m**). The solution to the MME is the BLUE of *β* and the BLUP of **m**. Henderson’s derivation assumes that **m** and **e** are normally distributed and maximizes the joint likelihood of (**y**, **m**) over the unknowns *β* and **m**. Maximizing the likelihood implies an optimization criterion of (**y** − **X***β* − **Zm**)′**R**^−1^(**y** − **X***β* − **Zm**) + **m**′**G**^−1^**m**, and it can be viewed as the “ridge regression formulation” of the BLUP (Ruppert *et al.* 2003).

We have assumed that *R* and *G* are known covariance matrices. In general, they are unknown and need to be estimated together with *β*, **m**. The REML approach to estimate the variance components maximizes the “restricted” likelihood associated with a specific set of linear combinations of the data. The restricted likelihood depends only on the variance components. REML produces unbiased estimates of the variance parameters *R* and *G*. More information about variance estimation using REML can be found in works by Corbeil and Searle (1976), Harville (1976), and McGilchrist (1993). There are other ways to derive the BLUP solution for **m**. Robinson (1991) showed that that the BLUE solution to *β* can be written as: $\hat{\beta }={\left(X\prime V^{-1}X\right)}^{-1}X\prime V^{-1}y$, and the BLUP solution to **m** can be written as: $\hat{m}=GZ\prime V^{-1}\left(y-X\hat{\beta }\right)$.

### LASSO method:

To overcome the limitations of linear least squares, we can use the LASSO for GS. LASSO was first introduced by Tibshirani (1996), and Usai *et al.* (2009) first implemented it in GS using cross-validation. We can write the model for individual *i* as:

$$y_{i}=\sum_{j=1}^{p}X_{ij}m_{j}+e_{i},$$ (5)

where *i* = 1…*n* individual, *j* = 1…*p* marker position, *y_i_* is the phenotypic value for individual *i*, *X_ij_* is an element of the incidence matrix corresponding to individual *i* and marker *j*, *m_j_* is the marker effect for marker *j*, and *e_i_* is the random residual. The LASSO estimate of the marker effect is obtained by minimizing the residual sum of squares $\sum_{i=1}^{n}{\left(y_{i}-\sum_{j=1}^{p}X_{ij}m_{j}\right)}^{2}$ subject to the constraint of the sum of the absolute value of the marker effects being less than a constant *s*, *s* ≥ 0, and we can write it as: $\sum_{j=1}^{p}\left|m_{j}\right|\leq s$. This constraint shrinks some of the marker effects and sets some of them to zero. One of the major differences between LASSO and ridge regression is that in LASSO as we increase the penalty, more marker effects will shrink to zero and in ridge regression all parameters will be reduced but still remain nonzero.

The LASSO estimator of the regression coefficients $m_{j}^{\prime }s$ can be found by an algorithm that was first described by Tibshirani (1996) and used computational ideas from Lawson and Hansen (1974). First, we assume that the elements of the incidence matrix are standardized such that $\sum_{i=1}^{n}X_{ij}=0$ and $\sum_{i=1}^{n}X_{ij}^{2}=n$. Then, the algorithm describes a quadratic programming problem with 2*^p^* linear constrains, corresponding to the different signs for the regression coefficients $m_{j}^{\prime }s$. For example, if *P* = 3 then we have:

$$m_{1}+m_{2}+m_{3}\leq s$$

$$m_{1}+m_{2}-m_{3}\leq s$$

$$m_{1}-m_{2}+m_{3}\leq s$$

$$m_{1}-m_{2}-m_{3}\leq s$$

$$-m_{1}+m_{2}+m_{3}\leq s$$

$$-m_{1}+m_{2}-m_{3}\leq s$$

$$-m_{1}-m_{2}+m_{3}\leq s$$

$$-m_{1}-m_{2}-m_{3}\leq s.$$

Let $f\left(m\right)=\sum_{i=1}^{n}{\left(y_{i}-\sum_{j=1}^{p}m_{j}X_{ij}\right)}^{2}$ and for *k* = 1…2*^p^* let *γ_k_* be a vector of indicator variables 1, 0, −1 depending on the signs of the regression coefficients corresponding to the *k^th^* inequality. Also, let $E=\left\{i:\gamma_{i}^{\prime }m=s\right\}$ and $S=\left\{i:\gamma_{i}^{\prime }m<s\right\}$. $G_{E}={\left[\gamma_{1},\gamma_{2},\ldots \gamma_{2^{p}}\right]}^{\prime }$.

The steps of the algorithm finding the LASSO estimator can be written as:

1. Let *E* = {*i*_0_}, where *i*_0_ corresponds to the least squares estimate of **m** and $\gamma_{i_{0}}=$ sign$\left({\hat{m}}_{LS}\right)$.
2. Find $\hat{m}$ such that f(m) is minimized subject to $G_{E}m\leq s1$.
3. If $\sum_{j=1}^{p}\left|m_{j}\right|\leq s$, then done.

If $\sum_{j=1}^{p}\left|m_{j}\right|>s$, $E=\left\{i_{0},i\right\}$ such that $\gamma_{i}=\mathrm{sign}\left(\hat{m}\right)$. Repeat steps 2 and 3.

The algorithm described above is computationally intensive. Efron *et al.* (2004) proposed a new model selection algorithm called least angle regression (LARS) that can be used in combination with LASSO estimation. LARS is similar to the traditional forward selection method. It starts with all the coefficients (marker effects) at zero. First, the marker that has the highest correlation with the phenotypic values is added into the model. The next marker added has to have a correlation with the residual that is at least as large. The third marker entered into the model is equiangular with the first two markers already in the model. At each iteration, a new marker is added, and the algorithm is accomplished in *p* iterations where *p* is the number of the available markers. However, for LASSO, the LARS procedure is modified. Because the LASSO has a constraint, the LARS procedure has to apply a restriction, so this model selection method is more closely related to the stepwise selection method. For a detailed description of LARS and the LARS–LASSO relationship, the reader can refer to Efron *et al.* (2004).

One other important question is how to find the upper bound of the sum of the absolute value of the marker effects, *s*. Finding the best value for *s* can be viewed as the selection of the size of the best subset of markers. Usai *et al.* (2009) used the cross-validation approach of Kohavi (1995) with random subsampling replication. In every replication, the data are randomly divided into a training set and a validation set. The training set is used to estimate the marker effects using the LARS algorithm for the LASSO method. The estimated marker effects were used to calculate the genomic breeding values (GEBV) for the individuals in the validation set, and then the correlation coefficients between the GEBV and the true phenotypic value were reported. The LARS iterations were carried forward until the maximum correlation was reached.

### Bayesian alphabet:

Meuwissen *et al.* (2001) proposed two hierarchical Bayesian models for GS denoted by Bayes A and Bayes B. In both methods the data and the variances of the marker positions need to be modeled. For individual *i* we can write:

$$y_{i}=\mu +\sum_{j=1}^{p}X_{ij}m_{j}+e_{i},$$ (6)

where *i* = 1…*n* individual, j=1...p marker position/segment, $y_{i}$ is the phenotypic value for individual *i*, *μ* is the *n* × 1 dimensional overall mean vector, *X_ij_* is an element of an incidence matrix for marker *j* and individual *i*, *m***_j_** is a random effect for marker *j*, and $e_{i}$ is a random residual. In general the model can be written as: $y=\mu +\sum_{j=1}^{p}X_{j}m_{j}+e$.

Inferences about model parameters are based on the posterior distribution. By Bayes’ Theorem, the posterior is obtained by combining the prior distribution and the likelihood function. For detailed information about Bayesian methods, the reader can refer to Kruschke (2010) or Gelman *et al.* (2003).

The difference between Bayes A and Bayes B lies in the way in which we model the variances of parameters. In both methods each marker position has its own variance. The Bayes A approach applies the same prior distribution for all of the variances of the marker positions. The scaled inverted (or inverse) chi-squared probability distribution χ^−2^(*ν*, *S*^2^) can be used with degrees of freedom *ν* and scale parameter *S*^2^ as the prior distribution. This is a convenient choice because it is a conjugate prior so the posterior distribution is in the same family of distributions as the prior distribution. The posterior distribution is also a scaled inverse chi-square distribution $\chi^{-2}\left(\nu +n_{j},S^{2}+m_{j}^{\prime }m_{j}\right)$ where *n_j_* is the number of haplotype effects at marker position *j*.

The Bayes B approach seems more realistic for GS than Bayes A. The only difference between the two methods is the prior for the variance components. Bayes B assumes that not all markers contribute to the genetic variation. It has a prior density on the variance that is a mixture. It has a high probability mass at *σ_mj_* = 0 and an inverted chi-square distribution when *σ_mj_* > 0. It can be summarized as *σ_mj_* = 0 with prob= *π* and *σ_mj_* ∼ *χ*^−2^(*ν*, *S*) with prob=(1 − *π*).

For the Bayes B method if *m*′*m* > 0, then one cannot sample $\sigma_{gj}^{2}=0$. So, we can sample *m_j_* and $\sigma_{gj}^{2}$ simultaneously by $p\left(\sigma_{mj}^{2},m_{j}|y^{*}\right)=p\left(\sigma_{mj}^{2}|y^{*}\right)p\left(m_{j}|\sigma_{mj}^{2},y^{*}\right)$, where $y^{*}$ is the data that are corrected for the mean and for all genetic effects except *m_j_*.

To sample from the distribution$p\left(\sigma_{mi}^{2}|y^{*}\right)$, we can use the Metropolis-Hastings algorithm in the following way:

1. Sample $\sigma_{m\left(new\right)}^{2}$ from the prior distribution of $p\left(\sigma_{mj}^{2}\right)$.
2. $\sigma_{mj}^{2}=\sigma_{m\left(new\right)}^{2}$ with probability of $Min\left[\frac{p\left(y^{*}|\sigma_{m\left(new\right)}^{2}\right)}{p\left(y^{*}|\sigma_{mj}^{2}\right)};1\right]$.

Using simulated data, it was shown that the Bayesian methods perform better in terms of prediction accuracy than the linear least squares regression, the ridge regression, and the BLUP method (Meuwissen *et al.* 2001; Habier *et al.* 2009, 2010). However, as Gianola *et al.* (2009) pointed out, the choice of the degrees of freedom and the scale parameters of the scaled inverse chi-square distribution can influence the outcome. Improved Bayesian methods were developed by Habier *et al.* (2011) to deal with the weakness of Bayes A and Bayes B. Bayes C uses a common variance for all SNPs, and for Bayes D the scale parameter of the scaled inverse chi-square distribution is estimated instead of specified by the user. Bayes C*π* and Bayes D*π* (Habier *et al.* 2011) are the modification of Bayes C and Bayes D where the probability of having a zero effect SNP *π* is estimated.

### Bayesian LASSO:

Park and Casella (2008) introduced the Bayesian LASSO method for estimating the regression coefficients. They used an idea from Tibshirani (1996) to connect the LASSO method with the Bayesian analysis. Tibshirani (1996) noticed that the LASSO estimates of the regression coefficients can be viewed as posterior mode estimates assuming that the regression coefficients have double exponential prior distributions. The Bayesian LASSO is also used in GS (de los Campos *et al.* 2009, 2010a; Long *et al.* 2011) using the hierarchical model with the likelihood function:

$$f\left(y|\mu ,X,m,\sigma^{2}\right)∼N\left(\mu +Xm,\sigma^{2}I\right),$$ (7)

where **y** is the *n* × 1 data vector, *μ* is the overall mean vector, **m** is a vector of the marker effects, and **X** is the design matrix that connects **m** to **y**. *N*(*μ* + **Xm**, *σ*^2^**I**) denotes the normal density with mean *μ* + **Xm** and variance *σ*^2^**I** where **I** is an *n* × *n* identity matrix. The prior distribution on the marker effects $m_{j}^{\prime }s$ *j* = 1…*p* can be written as $p\left(m_{j}|\tau_{j}^{2}\right)∼N\left(0,\tau_{j}^{2}\right)$, and the prior distribution on *τ_j_* is *p*(*τ_j_*|*λ*) ∼ *Exp*(*λ*) where *Exp*(*λ*) denotes the exponential distribution with rate parameter *λ*. Park and Casella (2008) and de los Campos *et al.* (2009) presented the full conditional distributions that were used to sample via the Gibbs sampler. de los Campos *et al.* (2009) expanded the model and assigned a prior distribution to *λ*^2^. The prior has a Gamma distribution with shape parameter *α*_1_ and scale parameter *α*_2_, and it can be written as *p*(*λ*^2^) ∼ Γ (*α*_1_, *α*_2_); *λ* has two interpretations. In the Bayesian formulation, it is the rate parameter that controls the shape of the prior distribution of the $\tau_{j}^{\prime }s$. In the LASSO setting, *λ* controls the penalty for minimizing the MSE.

## Nonparametric methods in genome-wide selection

In this section, we review some of the nonparametric estimation methods that have been proposed for the case where the form of the relationship between a response variable and a set of predictors is unknown. A popular approach, at least in terms of usage, is based on the kernel method proposed by Silverman (1986) in the context of density estimation. In that context, the goal is to estimate the unknown density using a smooth curve (Schucany 2004). The kernel method is the most commonly used nonparametric estimation procedure.

The kernel density estimator $\hat{f}\left(x\right)$ can be written in the form:

$$\hat{f}\left(x\right)=\frac{1}{nh}\sum_{i=1}^{n}K\left(\frac{x-X_{i}}{h}\right),$$

where *n* is the number of observations, *K* is the kernel function that satisfies the condition ∫K(x)dx=1, *h* is positive real-valued smoothing parameter (also called window width or bandwidth), *x* is the focal point, and *X_i_* is the *p* × 1 dimensional vector of dummy covariates for observation *i*. We can calculate $\hat{f}\left(x\right)$ at several focal points *x*, and the observations that are closer to the focal point will get a higher weight in the calculation, so the kernel function $K\left(\frac{x-X_{i}}{h}\right)$ gives bigger weight to observations closer to the focal point. The kernel function *K* is usually chosen to be a symmetric unimodal density, so the kernel density estimator $\hat{f}\left(x\right)$ is also a density. A commonly used kernel function is the Gaussian kernel given by:

$$K\left(\frac{x_{i}-x}{h}\right)=\frac{1}{{\left(2\pi \right)}^{p/2}}\exp\left[-\frac{1}{2}{\left(\frac{x_{i}-x}{h}\right)}^{\prime }\left(\frac{x_{i}-x}{h}\right)\right].$$

**Figure 1:**
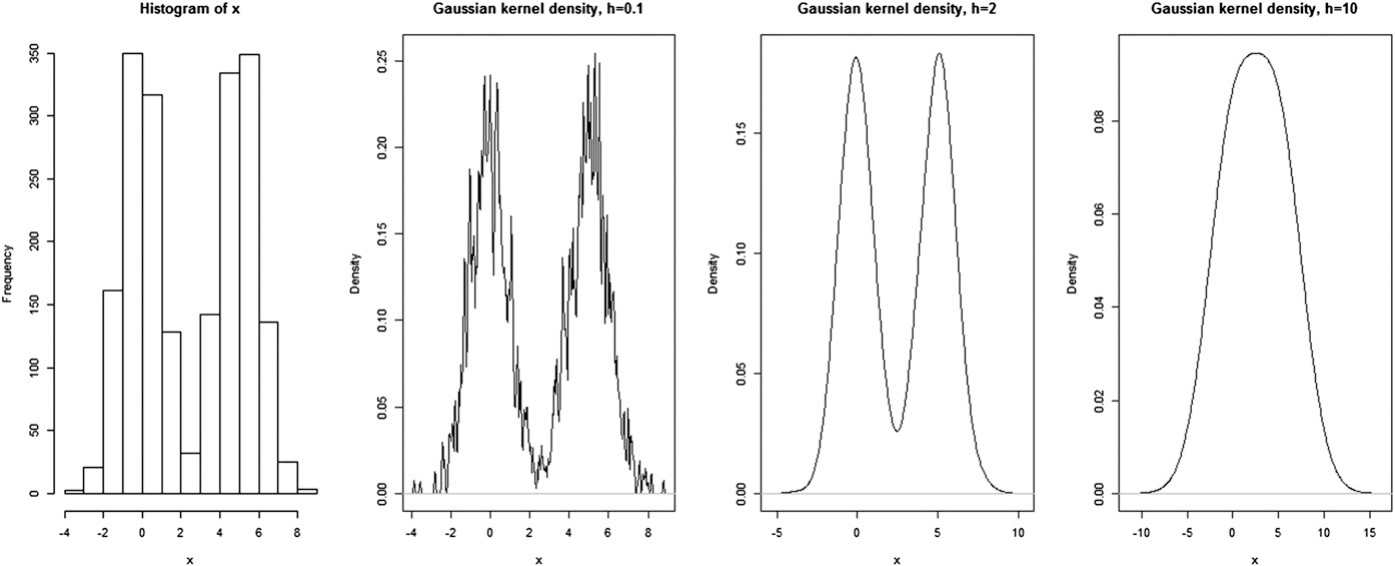
The influence of the bandwidth in kernel density estimation. From left to right, the first plot shows simulated data from a mixture of two normal distributions. The second, third, and fourth plots show the Gaussian kernel density estimates using bandwidth values *h* = 0.1, *h* = 2, and *h* = 10.

In this expression, observations with **x***_i_* coordinates closer to the focal point **x** are weighted more strongly in the computation of the fitted value $\hat{E}\left(y|x\right)$. The window width provides information about the range of observations that are included (Sheather 2004). Figure 1 shows how the kernel density estimation changes with different bandwidth values. Using simulated data from a mixture of two normal distributions, the second, third, and fourth panels show how the estimation changes with the change of the bandwidth value.

When *h* = 0.1, the data have strong influence on the density estimate, resulting in little bias and large variability among estimates. It is called an undersmoothed estimate. As we increase the bandwidth value, the estimates become smoother. When *h* = 10, the spread is too big and even the bimodal feature of the data disappears, which implies that the estimate is oversmoothed. Setting the bandwidth too large results in a large bias with little variance.

### Nadaraya-Watson estimator:

In the context of GS, Gianola *et al.* (2006) considered the regression function:

$$y_{i}=g\left(x_{i}\right)+e_{i},$$ (8)

*i* = 1, 2, …, *n* where *y_i_* phenotypic measurement on individual *i*, **x***_i_* is a *p* × 1 vector of dummy SNP covariates observed on individual *i*, *g*(.) is some unknown function relating genotypes to phenotypes, *g*(**x***_i_*) = *E*(*y_i_*|**x***_i_*), and *e_i_* is a random residual effect for individual *i* where *e_i_* ∼ (0, *σ*^2^) and is independent of **x***_i_*.

The conditional expectation function can be written in the form:

$$g\left(x\right)=\frac{\int yp\left(x,y\right)dy}{p\left(x\right)}.$$

A nonparametric kernel estimator (Silverman 1986) can be used to obtain an estimate of *p*(*x*). The estimator has the form:

$$\hat{p}\left(x\right)=\frac{1}{nh^{p}}\sum_{i=1}^{n}K\left(\frac{x_{i}-x}{h}\right),\;\int_{\infty }^{-\infty }\hat{p}\left(x\right)\mathrm{dx}=1,$$

where **x***_i_* is the observed p-dimensional SNP genotype of individual *i*, *i* = 1, 2, …, *n*. Similarly,

$$\hat{p}\left(x,y\right)=\frac{1}{nh^{p+1}}\sum_{i=1}^{n}K\left(\frac{y_{i}-y}{h}\right)K\left(\frac{x_{i}-x}{h}\right).$$

Using these expressions, Nadaraya (1964) and Watson (1964) showed that the conditional expectation function can be written as:

$$\begin{array}{c} \hat{E}\left(y|x\right)=\hat{g}\left(x\right)\\ =\frac{\int y\hat{p}\left(x,y\right)dy}{\hat{p}\left(x\right)}\\ =\frac{\frac{1}{nh^{p}}\sum_{i=1}^{n}y_{i}K\left(\frac{x_{i}-x}{h}\right)}{\frac{1}{nh^{p}}\sum_{i=1}^{n}K\left(\frac{x_{i}-x}{h}\right)}\\ =\sum_{i=1}^{n}y_{i}w_{i}\left(x\right),\;w_{i}\left(x\right)=\frac{K\left(\frac{x_{i}-x}{h}\right)}{\sum_{i=1}^{n}K\left(\frac{x_{i}-x}{h}\right)}. \end{array}$$

The estimator is just a weighted sum of the observations *y_i_*, *i* = 1…*n* and is called the NWE.

The selection of the bandwidth, h, value is challenging. Hardle (1990) discussed several approaches to select *h*, including the leave-one-out cross-validation (CV), penalizing functions, and plug-in methods. Gianola *et al.* (2006) used the leave-one-out CV approach to select the bandwidth. In this approach, first exclude the *i^th^* observation (*y_i_*, **x**) and fit the model to the other *n* − 1 observations. Using the marker information, predict $\hat{g}\left(x_{i}|h\right)$. This is repeated for all *n* observations. The CV criterion is (Clark 1975):

$$CV\left(h\right)=\frac{\sum_{i=1}^{n}{\left[y_{i}-\hat{g}\left(x_{i}|h\right)\right]}^{2}}{n}.$$

The CV estimate of *h* is the value of *h* that minimizes CV(h).

### Reproducing kernel Hilbert space:

Gianola *et al.* (2006) proposed a semi-parametric kernel mixed model approach in which they combined the nice features of a nonparametric model (described above) with a mixed model framework. The model can be written as:

$$y_{i}=w_{i}^{\prime }\beta +z_{i}^{\prime }u+g\left(x_{i}\right)+e_{i},$$ (9)

where *i* = 1, 2, …, *n*, *β* is a vector of fixed unknown effects (*e.g.*, physical location of an individual), *u* is a *q* × 1 vector of additive genetic effects, $w_{i}^{\prime }$ and $z_{i}^{\prime }$ are known incidence vectors, *g*(**x***_i_*) is an unknown function of the SNP data and the vector of residuals, and *e* is assumed to have a $N\left(0,I\sigma_{e}^{2}\right)$ distribution. The vector containing additive genetic effects, *u* is distributed as $N\left(0,A\sigma_{u}^{2}\right)$, where $\sigma_{u}^{2}$ is the additive genetic variance and **A** is the additive relationship matrix.

The authors suggested two different methods for estimation in this model. The first strategy, denoted “Mixed Model Analysis,” consists of a two-step approach with a “corrected” data vector $y_{i}-g\left(x_{i}\right)=w_{i}^{\prime }\beta +z\prime u+e_{i}$ in the second step of the analysis. A Bayesian approach can also be used where one can draw samples from the pseudo posterior distribution $\left[\beta ,u,\sigma_{u}^{2},\sigma_{e}^{2}|y*\right]$, and then form semi-parametric draws of the total genetic value.

The other method they suggested is the “Random *g*(.) function” approach, where it is assumed that *β*, *u* are known. In this case:

$$\hat{g}\left(x|\beta ,u,y,h\right)=\hat{E}\left(y_{i}-w_{i}^{\prime }\beta -z_{i}^{\prime }u|x\right)=\sum_{k=1}^{n}w_{k}\left(x\right)\left(y_{k}-w_{k}^{\prime }\beta -z_{k}^{\prime }u\right),$$

and draws of *β*^(^*^j^*^)^, *u*^(^*^j^*^)^ can then be obtained from the distribution $\left[\beta ,u,\sigma_{u}^{2},\sigma_{e}^{2}|y*,h\right]$.

Finally, Gianola *et al.* (2006) discuss estimation in the RKHS mixed model. The set-up is similar to the mixed model approach, but estimation of model parameters is performed using a penalized sum of squares approach. As before, the model can be written as:

$$y_{i}=w_{i}^{\prime }\beta +z^{\prime }u+g\left(x_{i}\right)+e_{i},$$ (10)

where *i* = 1, 2, …, *n*. The penalized sum of squares is given by:

$$SS\left(g\left(x\right),h\right)=\sum_{i=1}^{n}{\left[y_{i}-w_{i}^{\prime }\beta -z_{i}^{\prime }u-g\left(x_{i}\right)\right]}^{2}+h\left\|g\left(x\right)\right\|,$$

where the penalty ‖*g*(*x*)‖ is a function of the second derivatives of *g*(*x*). The goal is to find *g*(*x*) that minimizes the penalized SS. Wahba (1990) showed that the minimizer can be written as:

$$g\left(.\right)=\alpha_{0}+\sum_{j=1}^{n}\alpha_{j}K\left(.,x_{j}\right),$$

where *K*(.,.) is the reproducing kernel.

### Support vector machine regression:

SVM was proposed by Vapnik and discussed by Cortes and Vapnik (1995). SVM is a supervised learning technique that was originally developed as a classifier. A training data set is used to develop a maximum margin classifier that produces the largest possible separation between two classes of observations. In the linearly separable case, if observations (*x_i_*) ∈ *R^p^*, then the separator is a hyper-plane in *R^p^*^−1^.

Because fitting a regression model essentially consists of finding an optimal projection of the observations on a lower-dimensional hyper-plane, the idea can be used to estimate the unknown regression function subject to restrictions. The reader can refer to Hastie *et al.* (2009), Steinwart and Christmann (2008), and Christianini and Shawe-Taylor (2000) for a review of SVM. SVM regression was adopted by Maenhout *et al.* (2007) and Long *et al.* (2011) for GS in plant breeding. A nice feature of SVM regression in plant breeding applications is that the relationship between the marker genotypes and the phenotypes can be modeled with a linear or nonlinear mapping function that takes samples from a predictor space to an abstract, multidimensional feature space (Hastie *et al.* 2009).

Suppose that we have a training sample *S* = {(**x***_i_*, *y_i_*), **x***_i_ ε R^n^*_,_ *y_i_ ε R*, *i* = 1…*n*}, where **x***_i_* is a *p* dimensional vector containing the genotypic values for the *p* markers for individual *i*, and *y_i_* is the phenotypic value for individual *i*. A model that describes the relationship between the phenotype and the genotype of an individual can be written as:

f(x)=b+wx, (11)

where *b* is a constant and *w* is a vector of unknown weights. The constant *b* reflects the maximum error we are willing to commit when estimating the weights *w*. We learn about the function *f*(*x*) by minimizing the expression $\lambda \sum_{i=1}^{n}L\left(y_{i}-f\left(x_{i}\right)\right)+\frac{1}{2}{\left\|w\right\|}^{2}$. *L*(.) denotes the loss function that measures the quality of the estimation. The regularization parameter *λ* quantifies the trade-off between the sparsity and the complexity of the model. Increasing *λ* implies a higher penalty on the error. The norm ‖**w**‖ of vector **w** is inversely associated with model complexity; by choosing *w* to minimize ‖**w**‖, we reduce model complexity.

There are many loss functions used for SVM regression. Some of the popular loss function choices include the squared loss, absolute loss, and the *ε*-insensitive loss. Here, we present these loss function formulations.

1. The squared loss function has the form *L*(*y* − *f*(*x*)) = (*y* − *f*(*x*))^2^. It scales the loss quadratically by the size of the error. Using this loss function indicates that outliers are also weighted quadratically, which requires the user to deal with the outliers before the regression analysis.
2. The absolute loss function has the form *L*(*y* − *f*(*x*)) = |*y* − *f*(*x*)|. The absolute loss function scales the loss linearly by the size of the error eliminating the difficulty of using data sets with outliers.
3. The *ε*-insensitive loss function has a form:
  $$L\left(y-f\left(x\right)\right)=\{\begin{array}{ll} 0 & \text{if}\left|y-f\left(x\right)\right|<\epsilon \\ \left|y-f\left(x\right)\right|-\epsilon & \text{otherwise} \end{array},$$

where *ε* determines the number of support vectors used in the regression function. By definition (Vapnik 1995; Vapnik and Vashist 2009), a support vector is a vector **x***_i_* that satisfies the equation *y_i_*(*w***x***_i_* + *b*) = 1. Increasing *ε* implies that fewer support vectors are used in the fitting. The *ε*-insensitive loss function ignores the errors in the regression that have size less than *ε*. When the error is greater than *ε*, the loss is |*y* − *f*(*x*)| − *ε*.

**Figure 2:**
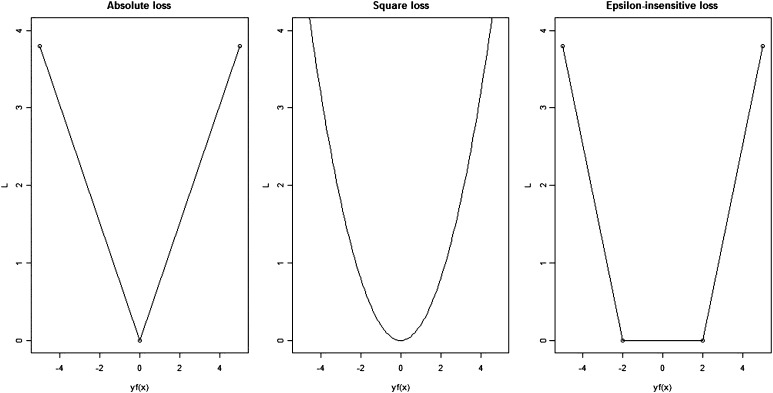
Loss functions used for SVM regression. The first panel shows the absolute loss function. The second panel is the square loss function, and the last panel is the *ε*-insensitive loss function.

Figure 2 illustrates the absolute loss, squared loss, and *ε*-insensitive loss functions as a function of the error *y* − *f*(*x*).

In the remainder, we focus on the *ε*-insensitive loss function, which needs a more robust representation to account for the noise in the data.

We can add extra “cost” (or allow for additional uncertainty) by introducing non-negative “slack variables” *ξ* constrained as follows (Long 2011):

*ξ*_1_*_i_* ≥ *y_i_* − *f*(**x***_i_*) − *ε*, where *i* = 1, …, *n n* is the number of training observations,

*ξ*_2_*_i_* ≥ *f*(**x***_i_*) − *y_i_* − *ε*, where *i* = 1, …, *n*.

We can now re-write the objective function to be minimized as:

$$\lambda \sum_{i=1}^{n}\left(\xi_{1i}+\xi_{2i}\right)+\frac{1}{2}{\left\|w\right\|}^{2}.$$

The solution to this constrained minimization problem has the form:

$$\hat{f}\left(x\right)=\sum_{i=1}^{n}\alpha_{i}x_{i}x+b$$

(Nocedal and Wright 1999). The solution depends on the training data through the inner product 〈**x***_i_*, **x***_j_*〉, which is a linear function of the observations.

To take advantage of higher dimensional feature spaces, we can introduce the data via nonlinear functions. For example, we can replace the inner product of the data by a kernel function:

$$k\left(x_{i},x_{j}\right)=\langle \phi \left(x_{i}\right),\phi \left(x_{j}\right)\rangle .$$

Some commonly used kernel functions include:

1. The linear kernel *k*(**x**, **z**) = 〈**x**, **z**〉
2. The Gaussian radial basis function *k*(**x**, **z**) = exp(−*σ*‖**x** − **z**‖^2^), where *σ* is the bandwidth parameter
3. The Laplace radial basis function *k*(**x**, **z**) = exp(−*σ*‖**x** − **z**‖).

The solution to the minimization problem can also be written as a function of the kernel function. The resulting expression is $\hat{f}\left(x\right)=\sum_{i=1}^{n}\alpha_{i}k\left(x,x_{i}\right)+b$. The choice of the kernel function and of the tuning parameters *λ*, *ε*, and *σ* are not straightforward. Because optimizing SVMs is not the focus of this article, we refer the reader to Cherkassky and Ma (2004).

### Neural networks:

NNs represent a nonparametric prediction procedure that captures additivity and epistasis by being able to model linear and complex nonlinear functions. The original idea of NN came from the theory of how neurons in the human brain work and interact, and how the brain conducts computations. In the NN, every unit is analogous to a brain neuron and the connections between them are analogous to synapses (Hastie *et al.* 2009). The first introduction of NNs in the context of brain architecture was presented by Bain (1873) and James (1890). McCulloch and Pitts (1943) developed a mathematical model for NNs.

**Figure 3:**
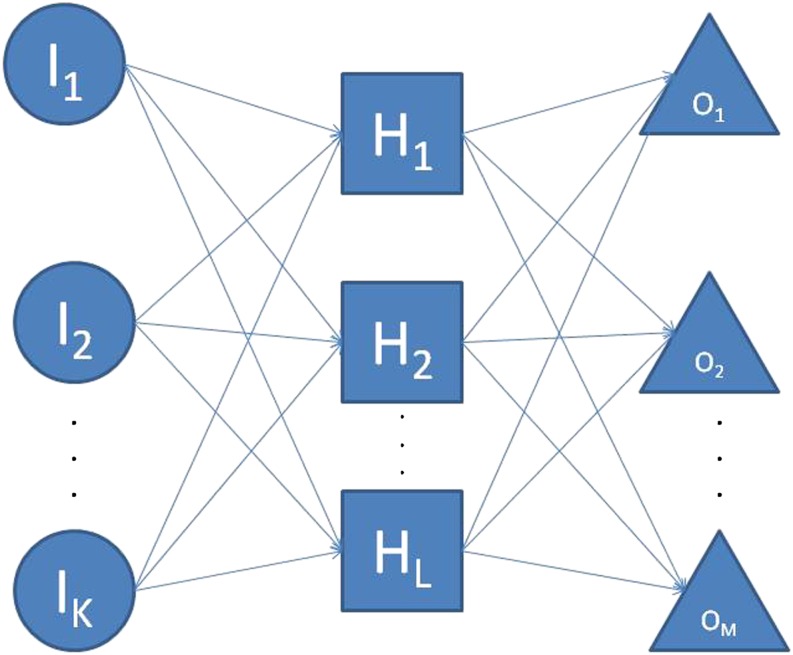
A three-layer feed-forward neural network with *K* input layer units, *L* hidden layer units, and *M* output layer units.

The basic layout of the NN is a two-stage network with three types of layers: an input layer; a hidden layer; and an output layer. This model is called the feed-forward NN and is illustrated in Figure 3.

Figure 3 shows a diagram of a three layer feed-forward NN with *K* input layer units, *L* hidden layer units, and *M* output layer units. *H*_1_, *H*_2_, …, *H_L_* are called hidden layer units because they are not directly observed. When the NN is used to estimate a regression function, there typically is only one output layer unit. The hidden layer units are functions of linear combinations of the inputs, and the output layer units are functions of the hidden layer units. The output function of a feed-forward NN can be expressed in the following form:

$$f\left(I_{k}\right)=\beta_{0}+\sum_{l=1}^{L}\beta_{l}\sigma \left(w_{l},b_{l},I_{k}\right),k=1,2,\ldots ,K,$$ (12)

where *K* is the number of units in the input layer, *I_k_* is the *k^th^* input, *β*_0_ *ε R^M^* is the intercept (bias terms), *M* is the number of output layer units, *L* is the number of hidden layer units, *β_l_* (*l* = 1, 2, …, *L*) are the output layer weights connecting the *l^th^* hidden layer unit to the output layer units, *σ* is the activation function modeling the connection between the hidden layer and the output layer, and *w_l_ ε R^K^* and *b_l_ ε R* are the unknown learning parameters of the hidden layer unit *l* (*l* = 1, 2, …, *L*) connecting the *k^th^* neuron in the input layer to them (Romero and Alquézar 2012).

In GS, typically *I_k_* represents a vector of predictors (marker genotypes or other information) collected on individual *k* (*k* = 1, 2, …, *K*), where K is the number of individuals in the analysis. The activation function *σ* is typically chosen to be the sigmoid (logistic) or the Gaussian radial basis function.

Gianola *et al.* (2011) implemented NNs for GS using two real data sets. Other examples are shown in work by Lampinen and Vehtari (2001) and Titterington (2004).

## Materials and Methods

For the purpose of illustrating the parametric and nonparametric prediction approaches, simulated data were created by the R (R Development Core Team 2008) package QTL Bayesian interval mapping (“qtlbim”) (Yandell *et al.* 2012). R can be downloaded from http://www.r-project.org, the qtlbim package can be accessed by library(qtlbim) in R, and the description of the package can be found at http://cran.r-project.org/web/packages/qtlbim/qtlbim.pdf. The reader can refer to Yandell *et al.* (2007) for detailed information about the qtlbim package. There are other publications as well where the qtlbim package is used to implement statistical methods. Some examples include Yi *et al.* (2007), Yi and Shriner (2008), and Piao *et al.* (2009). For comparing methods, we used a simulated *F*_2_ population with specifications listed in Table 1.

**Table 1:** Specification of the simulated *F*_2_ population: genetic architecture and heritability

Genetic Architecture	Heritability
Additive	0.70
Epistatic	0.70
Additive	0.30
Epistatic	0.30

We simulated four sets of phenotypic and genotypic information for a *F*_2_ and a BC population. The results for the BC population can be found in the supporting information section. For each set we created 20 replicates, which yielded to a total of 80 phenotypic and 80 genotypic data sets. Within each replicate we created 25 different training–testing data sets. Half of the data sets assume only additive effects and half assume only epistatic effects without any additive effects. We only evaluated the two extreme genetic architectures. Finally, for each genetic architecture, we generated data with two different narrow sense heritabilities. The low heritability was determined to be 0.30, and the high heritability was 0.70. For each of the simulated combinations of population, genetic architecture, and heritability, the data contain phenotypic information for 1000 individuals and genotypic information for 2000 biallelic markers (the possible values coded as “A” and “H”) for each individual. Out of the 1000 individuals, 800 were chosen randomly to be in the training set to fit the model, and 200 individuals were in the testing set. We predicted the phenotype for the individuals in the testing set. The qtlbim package uses Cockerham’s model as the underlying genetic model. The simulated genome has 10 chromosomes, each having a specified length. The 2000 markers were distributed throughout the genome in such a way that each chromosome had 200 markers and the markers were equally spaced over the chromosomes. We assumed no missing genotypic values and no missing phenotypic values. The phenotypic values are normally distributed.

For the additive model, we placed two QTL on each of the 10 chromosomes with either positive or negative additive effect. For the additive model we assumed no epistatic interaction.

**Figure 4:**
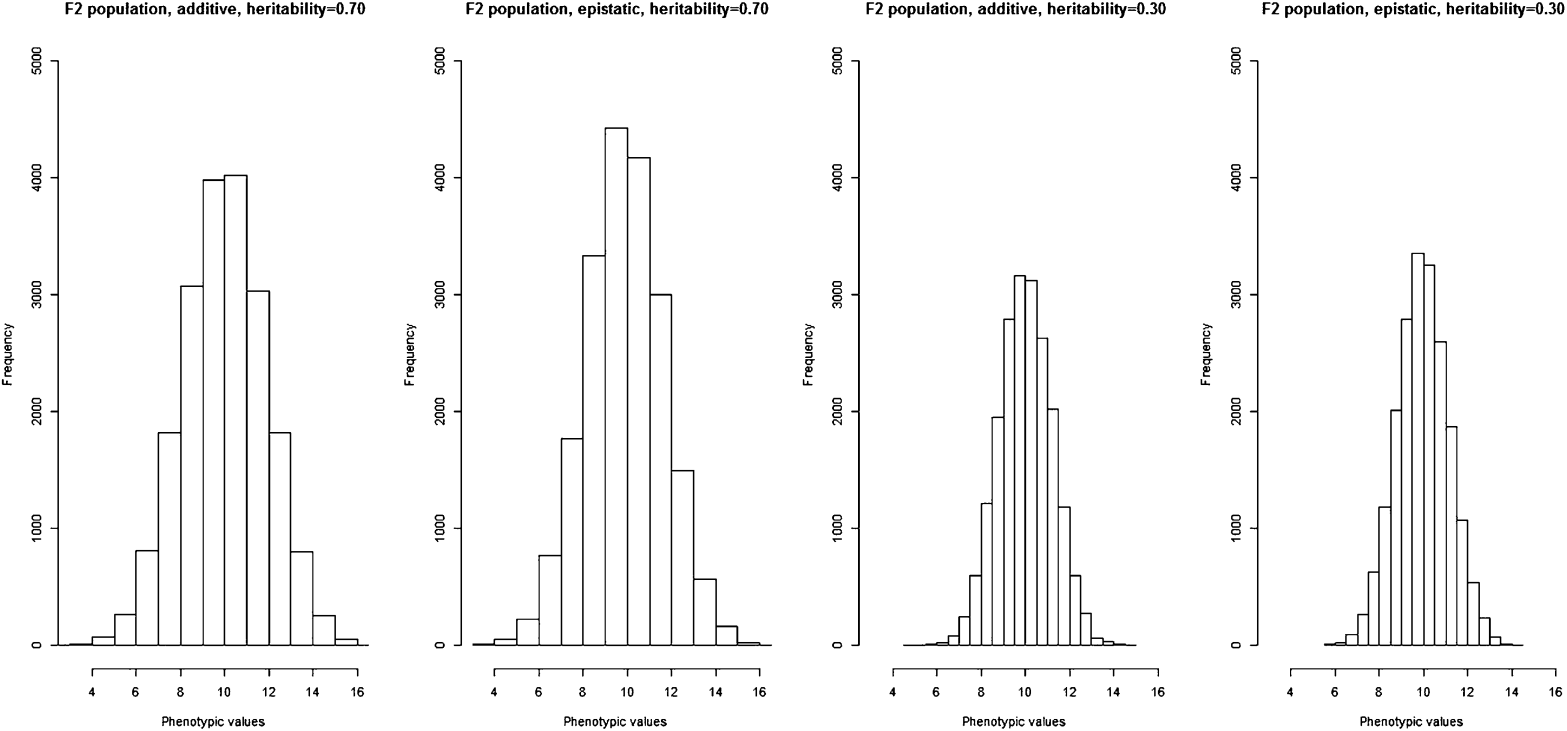
The histograms of the simulated phenotypic values. The histograms represent the distribution of the phenotypic values for the *F*_2_ population.

For the epistatic model, we only considered two-way interactions between the QTL. The interacting QTL were at the same genomic location as the QTL for the additive model, and only neighboring QTL were associated with each other, resulting in 10 two-way epistatic interactions, with each having either positive or negative epistatic effect on the phenotype. For the epistatic model, we assumed that the QTL contributed no additive effect. The phenotypic values were drawn from a normal distribution and are based on the *P* = *G* + *E* model. Figure 4 shows the histograms of the simulated phenotypic values for the four population–genetic architecture–heritability combinations.

To compare the performance of the methods, we used cross-validation, where we divided the data into training sets and testing sets. The training sets were used to fit the models, and the testing sets were used to determine the performance of the particular method. The performance of the methods was calculated by the accuracy of prediction and the MSE. We define accuracy of prediction as the correlation between the true phenotypic values and the predicted phenotypic values. We evaluated parametric methods including parametric least squares regression, ridge regression, Bayesian ridge regression, BLUP, LASSO, Bayesian LASSO, Bayes A, Bayes B, Bayes C, and Bayes C*π*. We also evaluated nonparametric methods, including NWE, RKHS method, SVM, and NN. To implement the parametric and nonparametric methods, the statistical software R and software written in C++ provided by the Animal Science Department at Iowa State University were used. Specifications of the parameters and inputs for each method are described below.

### Least squares regression

Because the number of markers exceed the number of the individuals in all simulated data sets, the idea of Meuwissen (2001) was adopted, and we first performed simple regression by coding each marker genotype as −1, 1. After fitting the 2000 simple linear models, one for each marker, we chose 300 of the markers with the most significant p-values. Then, these 300 markers were included into a final model, and we simultaneously used them to fit a linear model. To perform the linear regression, the *lm* function was used that can be found in the *stats* package (R Development Core Team 2008) in R. Finally, the prediction for the testing set was performed by using the marker data for the testing set and the output for the estimated marker effects provided by the *lm* function.

### Ridge regression

For this method, we used Gustavo de los Campos’ software written in R. The code implements the calculation of the ridge regression estimation of the marker effects discussed in the ridge regression section and uses these estimates to perform the prediction for the individuals in the testing set. For the procedure, all of the available phenotypic and marker information is used. In the estimation of the marker effect, the penalty parameter *λ* is chosen to have a value of: $\frac{1-h^{2}}{h^{2}}Var\left(X\right)$, where *h*^2^ is the narrow sense heritability and *Var*(*X*) is the sum of the 2000 marker variances. For all of the scenarios, we used *h*^2^ = 0.4.

### Bayesian ridge regression

To fit the Bayesian ridge regression model, the function Bayesian linear regression (BLR) was used, which can be found in the BLR package (de los Campos and Rodriguez 2010) in R. For the specifications of the BLR function, we used a Gaussian prior for the marker effects with mean 0 and a common variance $\sigma_{BR}^{2}$, where $\sigma_{BR}^{2}$ is unknown. $\sigma_{BR}^{2}$ is assigned to have a scaled inverse *χ*^2^ distribution with degrees of freedom *df_BR_* = 5 and scale parameter *S_BR_* = 0.01. The residual variance *σ_E_* has a scaled inverse *χ*^2^ distribution with degrees of freedom *df_E_* = 4 and scale parameter *S_E_* = 1. The BLR function implements the Gibbs sampler, and the number of iterations is specified to be 20,000. We used 2000 iterations for the burn-in period without any thinning. To fit the Bayesian ridge regression, we used all available phenotypic and genotypic data.

### BLUP

To implement BLUP, we used the *mixed.solve* function in R that can be found in the rrBLUP package (Endelman 2011). The available marker data were used as the design matrix for the random marker effects, and there was no fixed effect specified. The prediction was performed using the marker data for the testing set and the output for the predicted marker effects provided by the *mixed.solve* function.

### LASSO

To predict phenotypic values in the testing set using the LASSO method, we used the *glmnet* function of the *glmnet* package (Friedman *et al.* 2010) in R. For the initial parameter values, the default setting was applied. The prediction was performed with the tuning parameter, *λ*, that minimized the average cross-validation error (*cvm*).

### Bayesian LASSO

To fit the Bayesian LASSO method, the function BLR of the BLR package (de los Campos and Rodriguez 2010) in R was used. The regularization parameter, *λ*, is specified to be random and has a Gamma prior distribution with shape parameter *α*_1_ = 0.53 and rate parameter *α*_2_ = 0.00005. The residual variance *σ_E_* has a scaled inverse *χ*^2^ distribution with degrees of freedom *df_E_* = 4 and scale parameter *S_E_* = (*d_f_* − 2)(1 − *h*^2^)*Var*(*y*), where we specify that *d_f_* = 4, *h*^2^ = 0.5, and *Var*(*y*) is the phenotypic variance. The Gibbs sampler was applied with 20,000 iterations, and 2000 iterations were in the burn-in period. The chain was not thinned.

### Bayesian alphabet

To implement the Bayes A, Bayes B, Bayes C, and the Bayes C*π* models, software called *Gensel* (version 2.12) was used. GenSel was written by Fernando and Garrick (2008) in C++ and is used for GS in animal breeding populations. The software is not available for the public. However, it is available to Iowa State University research collaborators working on GS.

In GenSel Bayes A, Bayes B, Bayes C, and Bayes C*π* have been implemented. We used the settings for the four methods that are listed in Table 2.

**Table 2:** Parameter specifications for Bayes A, Bayes B, Bayes C, and Bayes C*π* used in GenSel

Parameters	Values
Chain length (number of iterations)	41,000
Burn-in period (number of iterations)	1000
Genotypic variance for data with *h*^2^ = 0.30	0.42
Genotypic variance for data with *h*^2^ = 0.70	2.10
Residual variance for data with *h*^2^ = 0.30	0.98
Residual variance for data with *h*^2^ = 0.70	0.90
Degrees of freedom for residual variance	10
Degrees of freedom for marker variance	4
*π*	0.70

The number of iterations used for chain length, burn-in period, the genotypic variance, the residual variance, the degrees of freedom for residual variance, the degrees of freedom for marker variance, and the probability corresponding to having a 0 effect marker are shown.

### Nadaraya-Watson estimator

To use the NWE for predicting the phenotypic values in the testing set, first we formed the cross-validation criteria and we evaluated it on a grid of values. We examined the cross-validation criteria between 1 and 1000 and chose the value to be the bandwidth, *h*, that minimized the criteria. Table 3 shows the bandwidth values that minimized each of the four data combinations for the NWE prediction. The code for calculating the optimal bandwidth value and for the prediction was written in R.

**Table 3:** Bandwidth values used for each of the four combinations of genetic architectures and heritabilities for the Nadaraya-Watson prediction

Genetic Architecture	Heritability	Bandwidth Value
Additive	0.70	195
Epistatic	0.70	195
Additive	0.30	205
Epistatic	0.30	205

### Reproducing kernel Hilbert space

The RKHS regression was based on methods and algorithms described by de los Campos *et al.* (2010b) and the R implementation was developed by Gustavo de los Campos *et al.* (2010b). To specify the RKHS regression, we chose the Gaussian reproducing kernel with the Euclidean distance for all of the eight combinations of genetic architectures, heritabilities, and population types. We fitted the model using three arbitrarily chosen bandwidth values. We performed the prediction for the testing set with each of the bandwidth values, and we averaged the three values of accuracy of selection and the three MSE values.

### Support vector machine

To implement the SVM regression, we used the *ksvm* function of the *kernlab* package (Karatzoglou *et al.* 2004) in R. For the *ksvm* function we used epsilon-regression as the type and the radial basis (Gaussian) kernel as the kernel function. After fitting the model, the *predict* function was used to perform the prediction of the phenotypic values for the testing set. For the other input parameters, the default values were used.

### Neural network

We implemented the NN model using the *brnn* function of the *brnn* package (Rodriguez and Gianola 2013) in R. This function fits a two-layer NN. We first map the input information into some basis function. Then, the inputs of the NN model are the marker-derived principal components. We specified the number of neurons to be three and the number of epochs to train to be 30 in the model. The other parameters were left at the default setting. For a detailed description of the application of the NN using the R package *brnn*, the reader can refer to Pérez-Rodiguez and Gianola (2013) and Pérez *et al.* (2013).

## Results and Discussion

We compared 10 parametric and four nonparametric statistical GS methods. Comparisons were based on predicted accuracies of a simulated F2 progeny derived from crosses of inbred lines where genotypic variability was responsible for either 30% or 70% of the phenotypic variability. The underlying genetic architectures responsible for the genotypic variability consisted of 20 independently segregating biallelic loci that contributed equally either in an additive manner to a quantitative phenotype or through additive by additive epistatic interactions among 10 pairs of loci. Each GS method was applied to 20 sets of simulated progeny with 25 replicates for each of the four combinations of genetic architecture and heritability, which yielded 500 total replicates for each combination. Training sets were used to develop a model, and the model was used to predict phenotypes in the testing sets. Training sets consisted of simulated phenotypes and 2000 marker genotypes for 800 random progeny while the testing sets associated with the training sets consisted of the same information for 200 progeny derived from the same cross. The accuracy of prediction was determined by calculating the correlation between the predicted phenotypic values for the 200 individuals in the testing set with the simulated phenotypic values for the same 200 individuals. The MSE values were determined by calculating the sum of the squared differences between the 200 predicted phenotypic values in the testing set and the 200 simulated phenotypic values, and then dividing the sum by 200.

**Figure 5:**
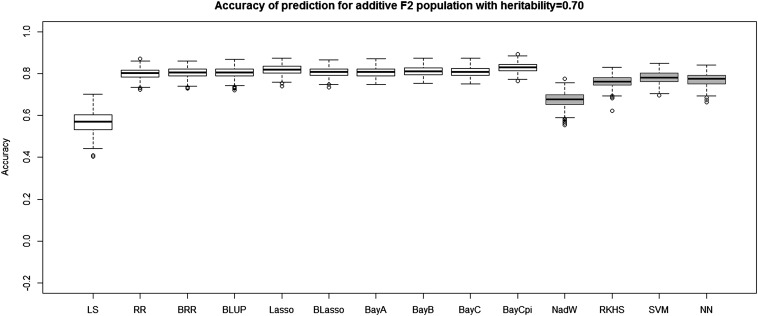
The boxplots of accuracy of prediction for the *F*_2_ population with additive genetic architecture and heritability of 0.70. The first 10 boxplots correspond to the parametric methods, and the last four (gray) boxplots correspond to the nonparametric methods.

**Figure 6:**
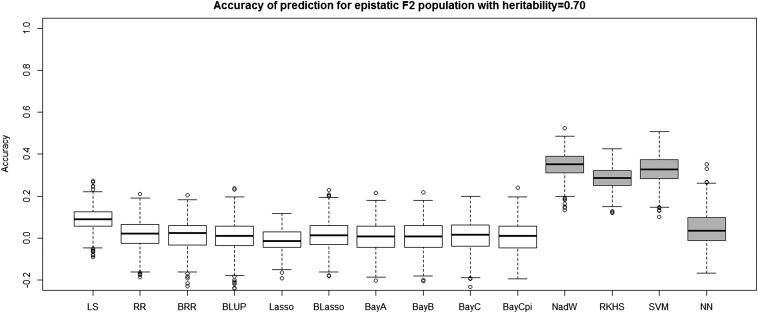
The boxplots of accuracy of prediction for the *F*_2_ population with epistatic genetic architecture and heritability of 0.70. The first 10 boxplots correspond to the parametric methods, and the last four (gray) boxplots correspond to the nonparametric methods.

**Figure 7:**
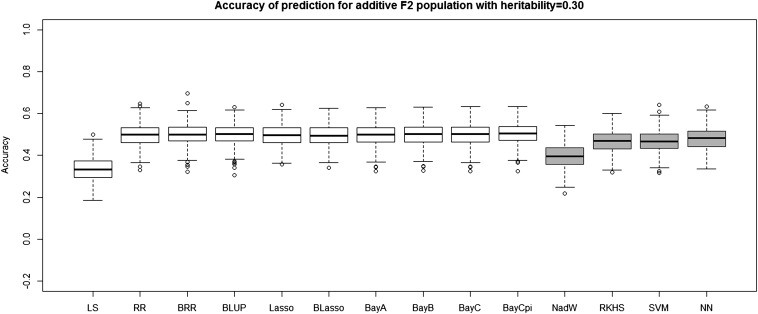
The boxplots of accuracy of prediction for the *F*_2_ population with additive genetic architecture and heritability of 0.30. The first 10 boxplots correspond to the parametric methods, and the last four (gray) boxplots correspond to the nonparametric methods.

**Figure 8:**
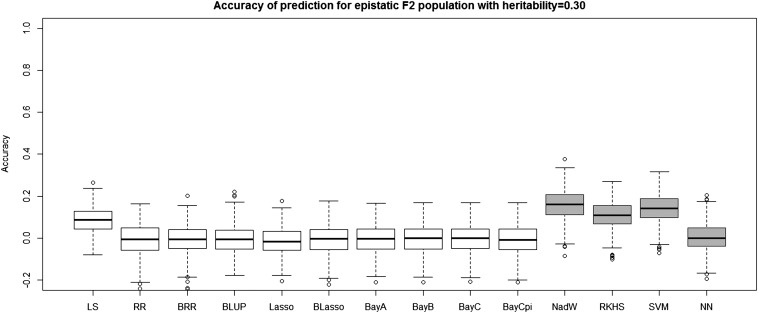
The boxplots of accuracy of prediction for the *F*_2_ population with epistatic genetic architecture and heritability of 0.30. The first 10 boxplots correspond to the parametric methods, and the last four (gray) boxplots correspond to the nonparametric methods.

**Figure 9:**
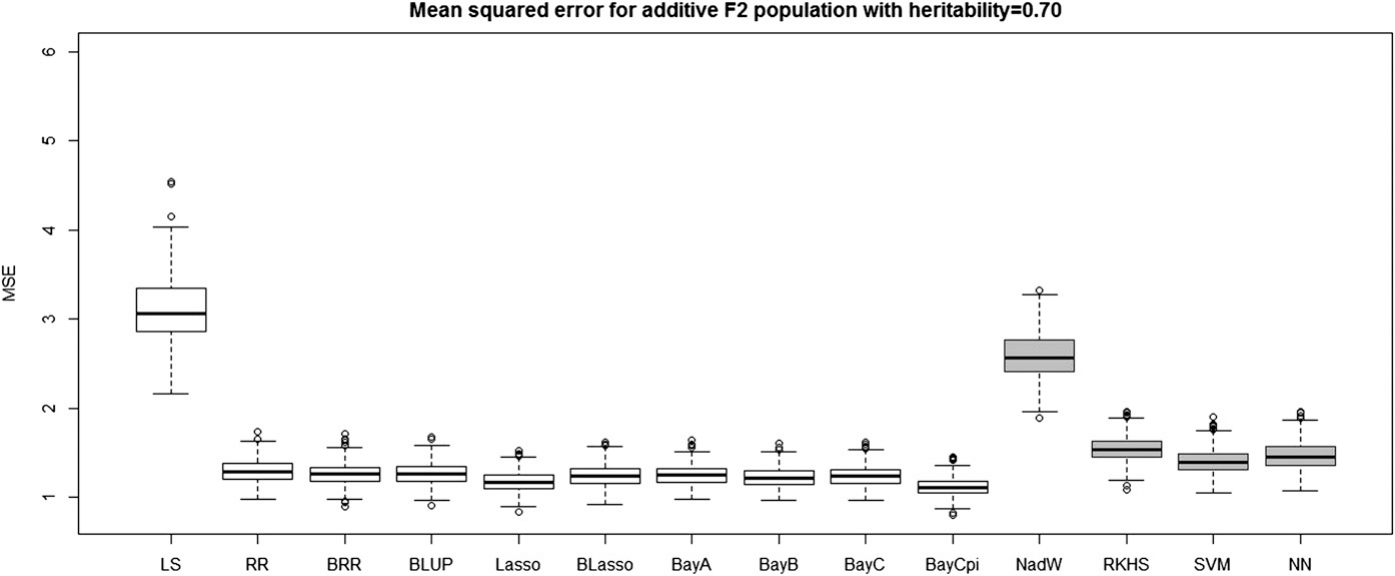
The boxplots of mean squared error for the *F*_2_ population with additive genetic architecture and heritability of 0.70. The first 10 boxplots correspond to the parametric methods, and the last four (gray) boxplots correspond to the nonparametric methods.

**Figure 10:**
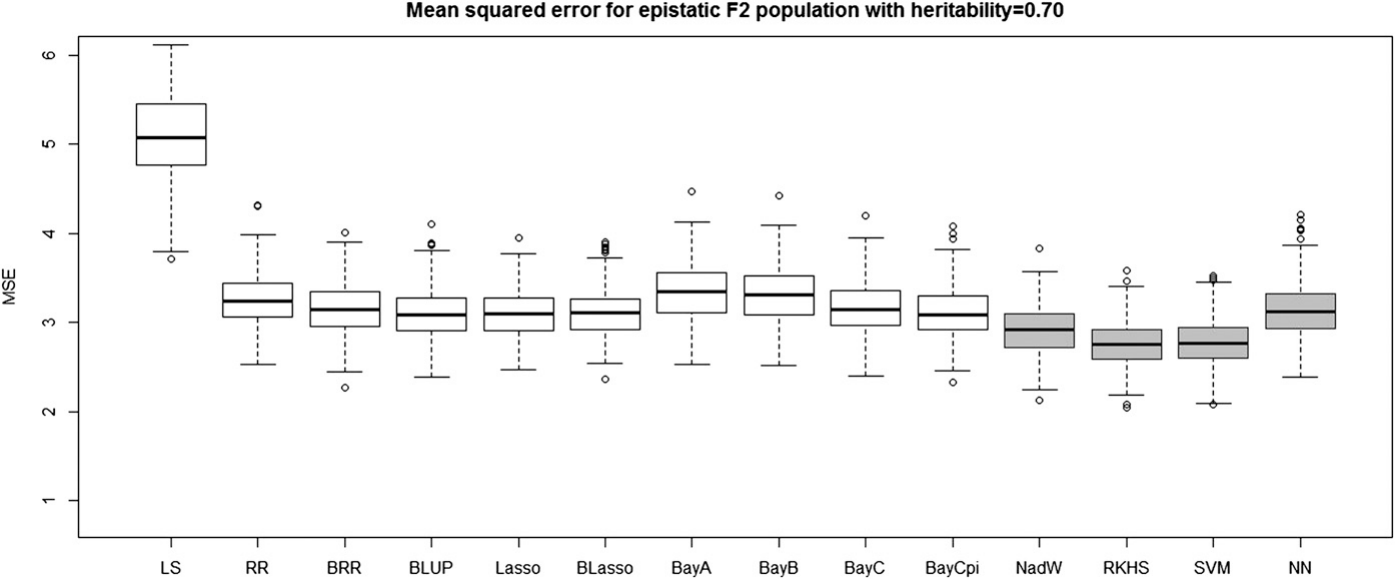
The boxplots of mean squared error for the *F*_2_ population with epistatic genetic architecture and heritability of 0.70. The first 10 boxplots correspond to the parametric methods, and the last four (gray) boxplots correspond to the nonparametric methods.

**Figure 11:**
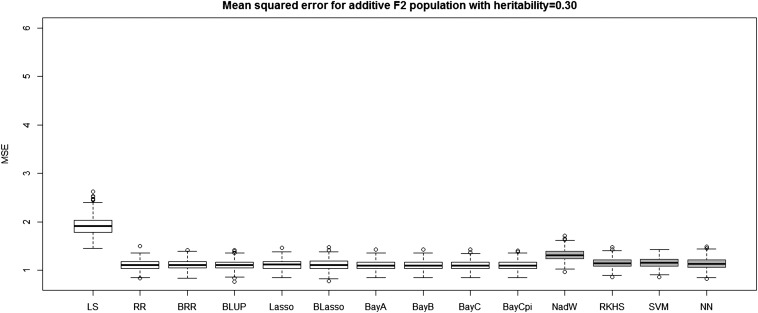
The boxplots of mean squared error for the *F*_2_ population with additive genetic architecture and heritability of 0.30. The first 10 boxplots correspond to the parametric methods, and the last four (gray) boxplots correspond to the nonparametric methods.

**Figure 12:**
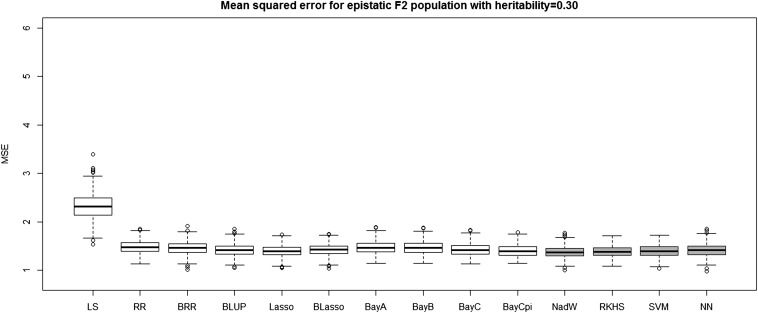
The boxplots of mean squared error for the *F*_2_ population with epistatic genetic architecture and heritability of 0.30. The first 10 boxplots correspond to the parametric methods, and the last four (gray) boxplots correspond to the nonparametric methods.

**Figure 13:**
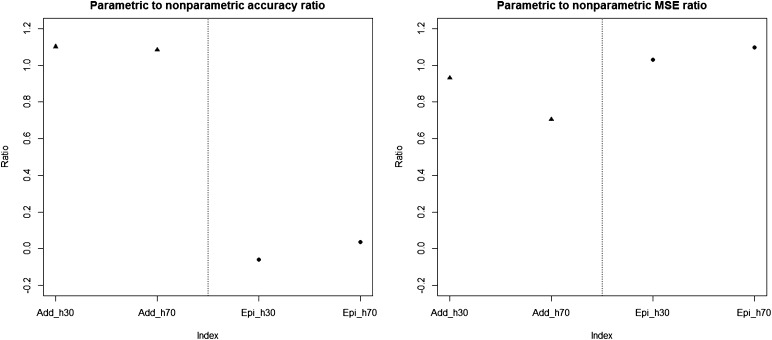
Plots of the parametric to nonparametric accuracy and MSE ratios. The left sides of the plots show the additive cases, and the right sides of the plots show the epistatic cases.

Table 4 and Table 5 report the average prediction accuracies and SE (sampling variabilities) of the 10 parametric and four nonparametric methods applied to the 500 replicates of the four combinations of genetic architecture and heritability. Table 6 and Table 7 report the average MSE values and SE of the MSE values of the 14 methods applied to the 500 replicates of the four combinations of genetic architecture and heritability. Figure 5, Figure 6, Figure 7, and Figure 8 each contain 14 boxplots of accuracy of prediction values for the 14 different methods. The boxplots show the distribution of the accuracy of prediction values for the 500 runs. Figure 9, Figure 10, Figure 11, and Figure 12 each contain 14 boxplots of MSE values for the 14 different methods. In each figure, the first 10 boxplots are for the parametric methods, and the last four (shaded) are for the nonparametric methods. These boxplots show the distribution of the MSE values for the 500 runs. The first plot of Figure 13 shows the ratio of the accuracy averaged over the parametric methods (excluding the least squares method because it is an outlier) and the accuracy averaged over the nonparametric methods, and the second plot of Figure 13 shows the ratio of the MSE averaged over the parametric methods (excluding the least squares method) and the MSE averaged over the nonparametric methods. The left sides of the plots show the ratios for the additive genetic architecture, and the right sides of the plots show the ratios for the epistatic genetic architecture. These summary plots clearly show the advantage of using nonparametric methods when epistasis is present. In both heritability scenarios, the parametric-to-nonparametric accuracy ratio is lower for the epistatic genetic architecture than for the additive genetic architecture. The parametric-to-nonparametric MSE ratio is higher for the epistatic genetic architecture than for the additive genetic architecture.

**Table 4:** Mean and SE of the prediction accuracy values for the parametric and the nonparametric methods for the *F*_2_ population with heritability *h*^2^ = 0.70

*F*_2_, *h*^2^ = 0.70, Accuracy	Additive Mean	Epistatic Mean	Additive SE	Epistatic SE
Least squares regression	0.56	0.09	0.05	0.06
Ridge regression	0.80	0.02	0.02	0.07
Bayesian ridge regression	0.80	0.01	0.02	0.07
BLUP	0.80	0.01	0.02	0.08
LASSO	0.82	−0.01	0.02	0.05
Bayes LASSO	0.81	0.01	0.02	0.07
Bayes A	0.81	0.00	0.02	0.07
Bayes B	0.81	0.01	0.02	0.07
Bayes C	0.81	0.01	0.02	0.07
Bayes C*π*	0.83	0.01	0.02	0.07
Nadaraya-Watson estimator	0.67	0.35	0.04	0.06
RKHS	0.76	0.29	0.03	0.05
Support vector machine	0.78	0.33	0.03	0.07
Neural network	0.77	0.05	0.03	0.09

Mean and SE of the prediction accuracy values for both the additive and the epistatic cases. The first 10 methods are parametric and the last four are nonparametric. The calculations for the epistatic mean and epistatic SE for the LASSO method are based on 213 replicates, for the epistatic mean and epistatic SE for the neural network method they are based on 493 replicates, and, for the rest, the calculations are based on 500 replicates.

**Table 5:** Mean and SE of the prediction accuracy values for the parametric and the nonparametric methods for the *F*_2_ population with heritability *h*^2^ = 0.30

*F*_2_, *h*^2^ = 0.30, Accuracy	Additive Mean	Epistatic Mean	Additive SE	Epistatic SE
Least squares regression	0.33	0.09	0.06	0.06
Ridge regression	0.50	−0.01	0.05	0.07
Bayesian ridge regression	0.50	−0.01	0.05	0.07
BLUP	0.50	−0.01	0.05	0.07
Lasso	0.50	−0.01	0.05	0.07
Bayes Lasso	0.50	0.00	0.05	0.07
Bayes A	0.50	0.00	0.05	0.07
Bayes B	0.50	0.00	0.05	0.07
Bayes C	0.50	0.00	0.05	0.07
Bayes C*π*	0.50	−0.01	0.05	0.07
Nadaraya-Watson estimator	0.40	0.16	0.05	0.07
RKHS	0.47	0.11	0.05	0.06
Support vector machine	0.47	0.14	0.05	0.07
Neural network	0.48	0.00	0.06	0.07

Mean and standard error of the prediction accuracy values for both the additive and the epistatic cases. The first 10 methods are parametric and the last four are nonparametric. The calculations for the epistatic mean and epistatic SE for the LASSO method are based on 184 replicates, for the epistatic mean and epistatic SE for the neural network method they are based on 498 replicates, and, for the rest, the calculations are based on 500 replicates.

**Table 6:** Mean and standard error of the mean squared error values for the parametric and the nonparametric methods for the *F*_2_ population with heritability *h*^2^ = 0.70

*F*_2_, *h*^2^ = 0.70, MSE	Additive Mean	Epistatic Mean	Additive SE	Epistatic SE
Least squares regression	3.10	5.10	0.36	0.53
Ridge regression	1.30	3.24	0.12	0.29
Bayesian ridge regression	1.27	3.14	0.13	0.29
BLUP	1.26	3.11	0.12	0.29
LASSO	1.17	3.10	0.11	0.26
Bayes LASSO	1.25	3.10	0.13	0.26
Bayes A	1.25	3.33	0.12	0.30
Bayes B	1.22	3.31	0.11	0.30
Bayes C	1.24	3.16	0.11	0.28
Bayes C*π*	1.11	3.11	0.11	0.27
Nadaraya-Watson estimator	2.59	2.91	0.25	0.26
RKHS	1.54	2.76	0.14	0.25
Support vector machine	1.40	2.76	0.14	0.26
Neural network	1.47	3.13	0.15	0.29

Mean and standard error of the prediction accuracy values for both the additive and the epistatic cases. The first 10 methods are parametric and the last four are nonparametric. The calculations are based on 500 replicates.

**Table 7:** Mean and standard error of the mean squared error values for the parametric and the nonparametric methods for the *F*_2_ population with heritability *h*^2^ = 0.30

*F*_2_, *h*^2^ = 0.30, MSE	Additive Mean	Epistatic Mean	Additive SE	Epistatic SE
Least squares regression	1.92	2.32	0.20	0.26
Ridge regression	1.11	1.48	0.10	0.13
Bayesian ridge regression	1.11	1.46	0.10	0.13
BLUP	1.11	1.42	0.10	0.12
LASSO	1.11	1.40	0.10	0.12
Bayes LASSO	1.11	1.42	0.11	0.12
Bayes A	1.10	1.47	0.10	0.13
Bayes B	1.10	1.46	0.10	0.13
Bayes C	1.10	1.42	0.10	0.13
Bayes C*π*	1.10	1.40	0.10	0.12
Nadaraya-Watson estimator	1.32	1.38	0.12	0.12
RKHS	1.15	1.39	0.10	0.12
Support vector machine	1.16	1.40	0.10	0.13
Neural network	1.14	1.41	0.11	0.12

Mean and standard error of the prediction accuracy values for both the additive and the epistatic cases. The first 10 methods are parametric and the last four are nonparametric. The calculations are based on 500 replicates.

Genetic architecture responsible for the genetic contribution to the phenotypes had the greatest impact on differences of accurate predictions among the GS methods. If the genetic architecture for the trait is due to additive-by-additive epistasis among 10 pairs of independently segregating loci, then parametric GS methods are unable to predict the phenotypes in the testing sets (shown in Figure 6, Figure 8, Figure 10, and Figure 12). In contrast, nonparametric methods, particularly the NWE, the RKHS, and SVM, provided predictions that are reasonably accurate, especially for traits with higher heritabilities (shown in Figure 6 and Figure 8). Our results are consistent with the statement by Gianola (2006) that nonparametric methods should be able to better predict phenotypes that are based on genetic architectures consisting of epistatic interactions. If the underlying genetic architecture is additive, then parametric GS methods are slightly better than the nonparametric methods for both levels of heritability and types of segregating progeny. Both the accuracy of prediction and the MSE results suggest the same about the models in terms of predictive performance. When additive effects are present, the least squares regression performs the worst among the parametric methods, and the NWE performs the worst among the nonparametric methods (shown in Figure 5, Figure 7, Figure 9, and Figure 11). When epistasis is present, the nonparametric NWE, the RKHS, and the SVM perform significantly better than the parametric methods (shown in Figure 6, Figure 8, Figure 10, and Figure 12). Among the parametric methods, the least squares regression has the highest accuracy of prediction values when epistasis is present. However, least squares has the highest MSE values among the parametric methods as well when epistasis is present. It suggests that the least squares method estimates the QTL effects from both loci involved in the epistasis more accurately than the other parametric methods in the *F*_2_ population. The parametric methods other than the least squares are shrinking the QTL effects too much. Among the nonparametric methods, the NN showed poor predictive ability when epistasis is present. We know that NN is prone to over-fitting (Lawrence *et al.* 1997; Smith 1996), which would affect prediction ability. Most of the results are consistent with the fact that parametric approaches assume that the explanatory variables fitted in the model are independent. When we only simulate additive effects, but not epistasis, the markers are assumed to be independent. In this case, we satisfy the parametric model assumption of having independent explanatory variables, so the parametric models have a larger predictive power than the nonparametric models. However, when we simulate epistasis, the markers are dependent, which violates the parametric model assumption. Nonparametric models can handle epistatic models without explicitly modeling the interactions.

Recently, the inability of parametric GS methods to predict has been observed in experimental data. Parametric GS methods were unable to predict chill coma recovery, a quantitatively measured adaptive trait in Drosophila (Trudy F. C. MacKay, personal communication). Two-dimensional scans of the whole genome had previously revealed that the genetic architecture of this trait is composed primarily of interactions involving many loci. Thus, the simulated architectures used in our study are reasonable for many quantitative traits.

The clear distinctions of estimated accuracies and MSE values between parametric and nonparametric methods when underlying genetic architecture is epistatic suggest that data analyses consisting of a combination of parametric and nonparametric GS methods could be used as a diagnostic to reveal the prevalent genetic architecture of the trait. It is likely that the true underlying genetic architecture consists of mixtures of additive and epistatic genetic effects, so the inferential limits of applying pairs of GS methods to data from samples of breeding populations as a diagnostic needs further investigation. However, the first step is to look at the extremes in terms of genetic architecture.

Our results also suggest that if the goal of the research is to accurately predict the genotypic value of an individual, particularly for purposes of selection, and if the underlying genetic architecture of the traits are not known, then it is best to use the nonparametric NWE, the RKHS, or the SVM. Unfortunately, these methods do not provide interpretable inferences about relative weighting that is being applied to various regions of the genome, *i.e.*, inferences about specific allelic contributions to the trait are limited. If the goal is genetic improvement and the underlying genetic architecture is known to be additive, then parametric GS methods will provide better predictions for selection. It has previously been hypothesized (Xu *et al.* 2011) that if all specific desirable alleles are known, then gene stacking (genome construction) based on optimization approaches will be more effective and efficient than GS approaches. Thus, in the interest of both immediate and long-term genetic improvement goals, a combination of data analyses consisting of parametric, nonparametric GS methods as well as genetic mapping (Guo *et al.* 2013) should be applied to data derived from plant breeding populations.

Although heritability did not affect the ability to distinguish among GS methods, it did affect estimated accuracies. When heritability is high and genetic architecture is additive, predictions are more accurate than for low heritability. When genetic architecture is based on epistasis and the trait exhibits low heritability, predictions are not very accurate for almost all GS methods. Even when the heritability is 0.70, the highest mean for prediction accuracy is 0.35, which indicates that further improvement of the models is necessary. Also, further research is needed to determine the affects of more complex plant breeding population structures. Typically, plant breeding population structures consist of inbred progeny derived from multiple crosses involving related and unrelated inbreds (Guo and Beavis 2011; Guo *et al.* 2013). Thus, GS needs to accurately predict phenotypes not only among subsets of progeny from related families within generations but also among generations of related and unrelated families.

In practice, plant breeders do not know the genetic architecture responsible for quantitative traits and the dynamics of selection for genetic improvement will tend to favor alleles that contribute to additive components. Genetic improvement is affected not only by the underlying genetic architecture but also by additional types of unpredictable genetic contributions including intra-locus dominance and genotype by environment interactions. Herein, we have demonstrated the superior ability of the nonparametric NWE, the RKHS, and the SVM methods to accurately predict phenotypes for additive by additive inter-locus interactions. We hypothesize that nonparametric GS methods also will enable more accurate predictions of individual genotypic value for traits that are affected by dominance and genotype by environment interactions.

## Supplementary Material

Supporting Information
