# Supplementary material for: Parametric and Nonparametric Statistical Methods for Genomic Selection of Traits with Additive and Epistatic Genetic Architectures
Source: G3 (Bethesda). 2014 Apr 9;4(6):1027–46. doi: 10.1534/g3.114.010298 (PMC4065247; doi:10.1534/g3.114.010298)
Supplement: Supporting Information [file supp_4_6_1027__index.html]

Parametric and Nonparametric Statistical Methods for Genomic Selection of Traits with Additive and Epistatic Genetic Architectures — Supporting Information 

# Parametric and Nonparametric Statistical Methods for Genomic Selection of Traits with Additive and Epistatic Genetic Architectures

## Supporting Information for Howard, Carriquiry, and Beavis, 2014

**Files in this Data Supplement:**

- File S1 - Supporting Information (PDF, 579 KB)
