# Supplementary material for: Parametric and Nonparametric Statistical Methods for Genomic Selection of Traits with Additive and Epistatic Genetic Architectures
Source: G3 (Bethesda). 2014 Apr 9;4(6):1027–46. doi: 10.1534/g3.114.010298 (PMC4065247; doi:10.1534/g3.114.010298)

G3 · Genes | Genomes | Genetics

Parametric and Nonparametric Statistical Methods for Genomic Selection of Traits with Additive and Epistatic Genetic Architectures

Réka Howard[*†1], Alicia L. Carriquiry[*], William D. Beavis[†]

[*]Department of Statistics, Iowa State University, Ames, Iowa, 50011, [†]Department of Agronomy, Iowa State University, Ames, Iowa, 50011

[1]Corresponding author: Réka Howard, Department of Statistics, Snedecor Hall, Ames, IA 50011.
Tel: 515-294-3440, E-mail: howardr@iastate.edu

DOI: 10.1534/g3.114.010298

File S1

The parametric and nonparametric methods were also evaluated on simulated backcross (BC) populations. The results for the simulated BC populations were similar to the results for the F_2 populations, so we are only providing the results for the BC populations as supporting information.

The specifications of the BC populations are given in Table 1. Figure 1 shows the histograms of the simulated phenotypic values for the 4 population - genetic architecture - heritability combinations. Table 2 shows the bandwidth values that minimized each of the 4 data combinations for the Nadaraya-Watson prediction. Tables 3-4 report the average prediction accuracies and standard errors (sampling variabilities) of the ten parametric and four non-parametric methods applied to the 500 replicates of the four combinations of genetic architecture and heritability. Tables 5-6 report the average MSE values and standard errors of the MSE values of the 14 methods applied to the 500 replicates of the four combinations of genetic architecture and heritability. Figures 2-5 each contain 14 boxplots of accuracy of prediction values for the 14 different methods. The boxplots show the distribution of the accuracy of prediction values for the 500 runs. Figures 6-9 each contain 14 boxplots of MSE values for the 14 different methods. In each figure, the first 10 boxplots are for the parametric methods, and the last 4 (shaded) are for the nonparametric methods. These boxplots show the distribution of the MSE values for the 500 runs. The first plot of figure 10 shows the ratio of the accuracy averaged over the parametric methods (excluding the LS method as it is an outlier) and the accuracy averaged over the nonparametric methods, and the second plot of Figure 10 shows the ratio of the MSE averaged over the parametric methods (excluding the LS method) and the MSE averaged over the nonparametric methods. The left side of the plots show the ratios for the additive genetic architecture, and the right side of the plots show the ratios for the epistatic genetic architecture.

There is little difference in estimated accuracies and MSE between the F_2 and BC progenies that is not accounted for by differences in genetic architecture and heritability. While there is little impact on accuracy of GS in these two types of progeny from a single cross of inbred lines, further research is needed to determine the affects of more complex plant breeding population structures.

Table 1. Specification of the simulated populations. The table contains information about the population type, the genetic architecture and the heritability.

Population type	Genetic architecture	Heritability
BC	additive	0.70
BC	epistatic	0.70
BC	additive	0.30
BC	epistatic	0.30

Figure 1. The histogram of the simulated phenotypic values. The histograms represent the distribution of the phenotypic values for the BC populations.

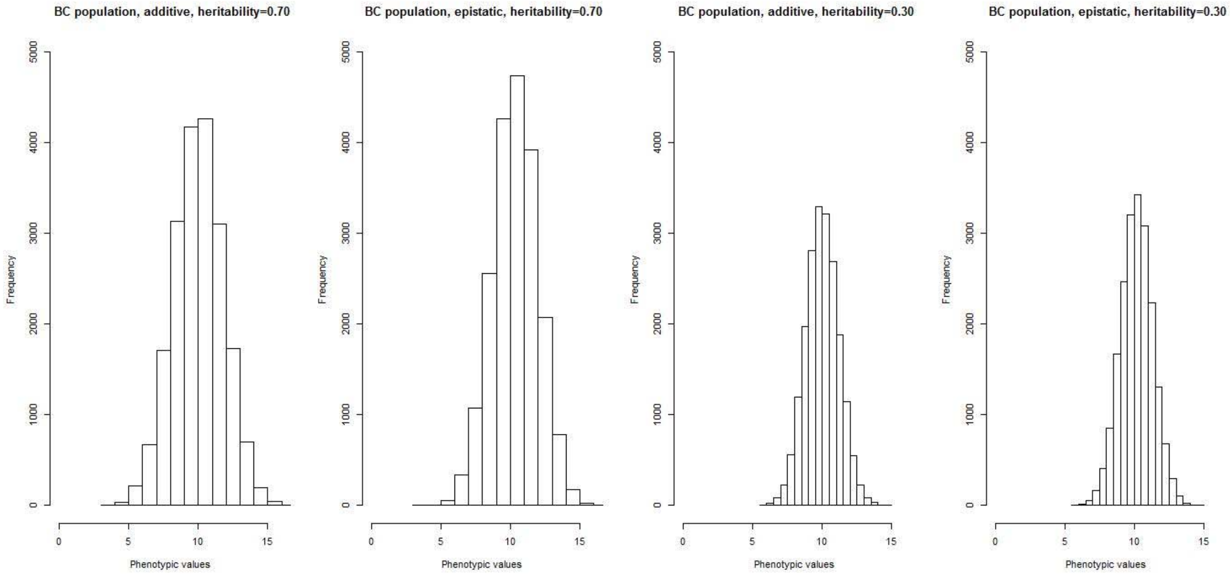

Table 2. Bandwidth values used for each of the eight combinations of genetic architectures, heritabilities and population types for the Nadaraya-Watson prediction.

Population type	Genetic architecture	Heritability	Bandwidth value
BC	additive	0.70	100
BC	epistatic	0.70	100
BC	additive	0.30	105
BC	epistatic	0.30	105

Table 3. The mean and standard error of the prediction accuracy values for the parametric and the nonparametric methods for the BC population with heritability h^2=0.70. The table contains the mean and standard error of the prediction accuracy values for both the additive and the epistatic cases. The first 10 methods are parametric, and the last 4 are nonparametric. The calculations for the epistatic mean and epistatic s.e. for the LASSO method are based on 183 replicates, for the epistatic mean and epistatic s.e. for the Neural Network method are based on 494 replicates, and for the rest, the calculations are based on 500 replicates.

BC, h^2=0.70, Accuracy	Additive mean	Epistatic mean	Additive s.e.	Epistatic s.e.
Least squares regression	0.53	0.08	0.05	0.06
Ridge regression	0.78	0.00	0.03	0.08
Bayesian Ridge regression	0.78	0.00	0.03	0.08
BLUP	0.78	0.01	0.03	0.08
LASSO	0.80	0.00	0.02	0.08
Bayes LASSO	0.79	0.00	0.03	0.07
Bayes A	0.78	0.00	0.03	0.08
Bayes B	0.79	0.00	0.03	0.08
Bayes C	0.79	0.00	0.03	0.08
Bayes Cπ	0.81	0.00	0.02	0.07
Nadaraya-Watson estimator	0.66	0.23	0.04	0.08
RKHS	0.77	0.18	0.03	0.07
Support Vector Machine	0.76	0.22	0.03	0.07
Neural Network	0.75	0.00	0.03	0.07

Table 4. The mean and standard error of the prediction accuracy values for the parametric and the nonparametric methods for the BC population with heritability h^2=0.30. The table contains the mean and standard error of the prediction accuracy values for both the additive and the epistatic cases. The first 10 methods are parametric, and the last 4 are nonparametric. The calculations for the epistatic mean and epistatic s.e. for the LASSO method are based on 201 replicates, for the epistatic mean and epistatic s.e. for the Neural Network method are based on 494 replicates, and for the rest, the calculations are based on 500 replicates.

BC, h^2=0.30, Accuracy	Additive mean	Epistatic mean	Additive s.e.	Epistatic s.e.
Least squares regression	0.32	0.08	0.06	0.06
Ridge regression	0.47	-0.01	0.05	0.08
Bayesian Ridge regression	0.47	-0.01	0.05	0.07
BLUP	0.47	0.00	0.05	0.07
LASSO	0.47	-0.01	0.06	0.07
Bayes LASSO	0.47	0.00	0.05	0.07
Bayes A	0.46	-0.01	0.05	0.08
Bayes B	0.47	-0.01	0.05	0.08
Bayes C	0.47	-0.01	0.05	0.08
Bayes Cπ	0.46	-0.01	0.05	0.07
Nadaraya-Watson estimator	0.38	0.09	0.06	0.07
RKHS	0.46	0.03	0.06	0.07
Support Vector Machine	0.45	0.07	0.05	0.07
Neural Network	0.46	0.00	0.06	0.07

Table 5. The mean and standard error of the mean squared error values for the parametric and the nonparametric methods for the BC population with heritability h^2=0.70. The table contains the mean and standard error of the prediction accuracy values for both the additive and the epistatic cases. The first 10 methods are parametric, and the last 4 are nonparametric. The calculations are based on 500 replicates.

BC, h^2=0.70, MSE	Additive mean	Epistatic mean	Additive s.e.	Epistatic s.e.
Least squares regression	3.07	4.52	0.38	0.49
Ridge regression	1.27	2.74	0.13	0.26
Bayesian Ridge regression	1.24	2.63	0.12	0.22
BLUP	1.24	2.58	0.12	0.21
LASSO	1.15	2.58	0.12	0.24
Bayes LASSO	1.22	2.59	0.13	0.23
Bayes A	1.27	2.88	0.13	0.25
Bayes B	1.23	2.83	0.13	0.25
Bayes C	1.22	2.65	0.13	0.22
Bayes Cπ	1.10	2.58	0.11	0.22
Nadaraya-Watson estimator	2.32	2.47	0.26	0.25
RKHS	1.32	2.46	0.13	0.22
Support Vector Machine	1.35	2.45	0.13	0.24
Neural Network	1.43	2.58	0.16	0.22

Table 6. The mean and standard error of the mean squared error values for the parametric and the nonparametric methods for the BC population with heritability h^2=0.30. The table contains the mean and standard error of the prediction accuracy values for both the additive and the epistatic cases. The first 10 methods are parametric, and the last 4 are nonparametric. The calculations are based on 500 replicates.

BC, h^2=0.30, MSE	Additive mean	Epistatic mean	Additive s.e.	Epistatic s.e.
Least squares regression	1.96	2.33	0.21	0.27
Ridge regression	1.09	1.41	0.10	0.12
Bayesian Ridge regression	1.10	1.38	0.10	0.13
BLUP	1.09	1.33	0.10	0.12
LASSO	1.09	1.35	0.10	0.12
Bayes LASSO	1.10	1.33	0.10	0.13
Bayes A	1.10	1.44	0.10	0.14
Bayes B	1.10	1.43	0.10	0.14
Bayes C	1.08	1.36	0.10	0.13
Bayes Cπ	1.10	1.34	0.11	0.12
Nadaraya-Watson estimator	1.26	1.32	0.11	0.12
RKHS	1.11	1.33	0.11	0.12
Support Vector Machine	1.13	1.37	0.11	0.13
Neural Network	1.12	1.34	0.11	0.12

Figure 2. The boxplots of accuracy of prediction for the BC population with additive genetic architecture and heritability of 0.70. The first 10 boxplots correspond to the parametric methods, and the last 4 (gray) boxplots correspond to the nonparametric methods.

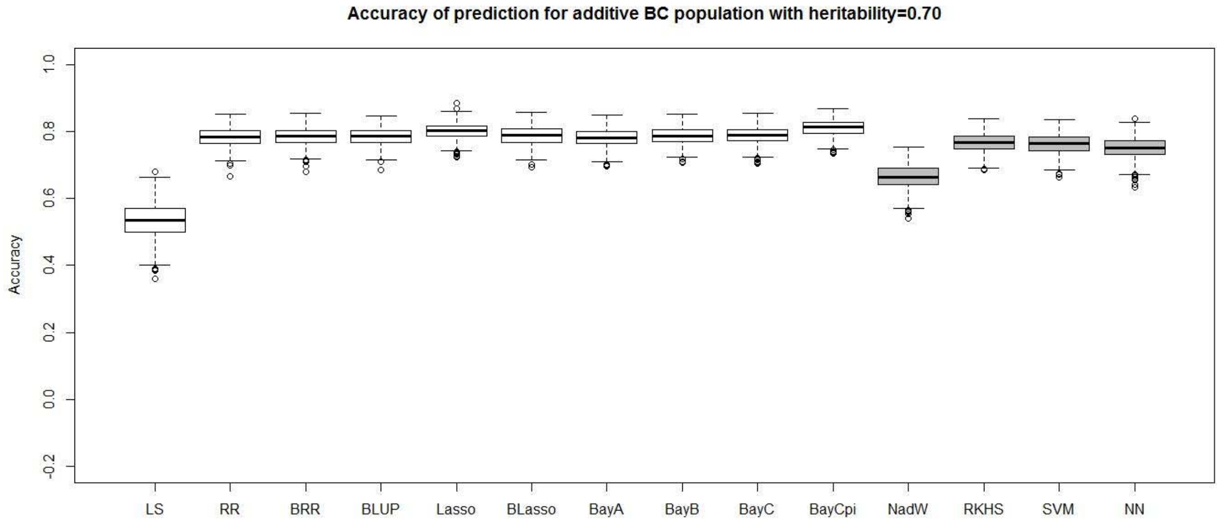

Figure 3. The boxplots of accuracy of prediction for the BC population with epistatic genetic architecture and heritability of 0.70. The first 10 boxplots correspond to the parametric methods, and the last 4 (gray) boxplots correspond to the nonparametric methods.

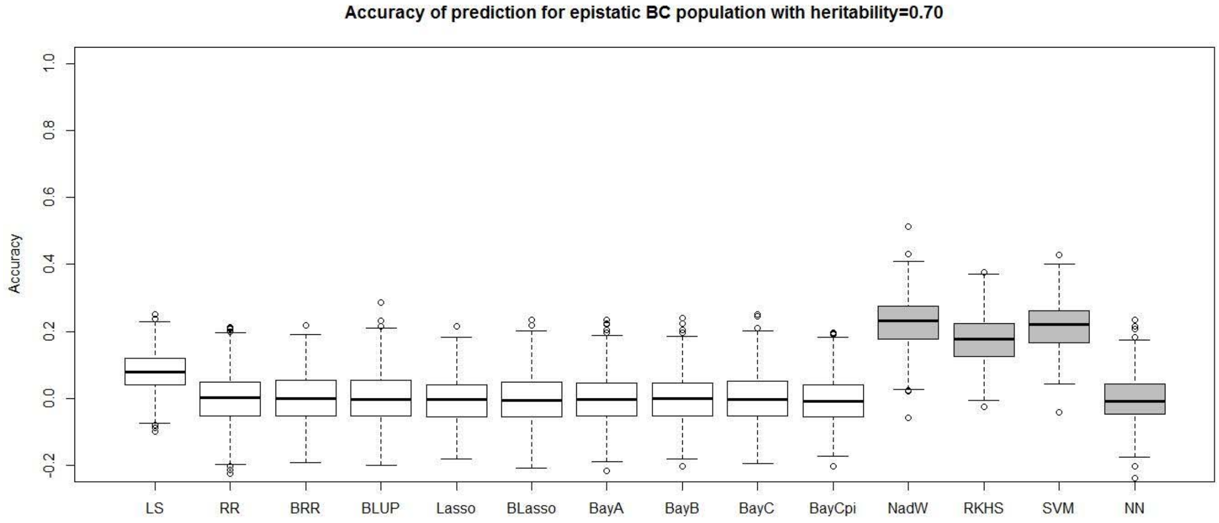

Figure 4. The boxplots of accuracy of prediction for the BC population with additive genetic architecture and heritability of 0.30. The first 10 boxplots correspond to the parametric methods, and the last 4 (gray) boxplots correspond to the nonparametric methods.

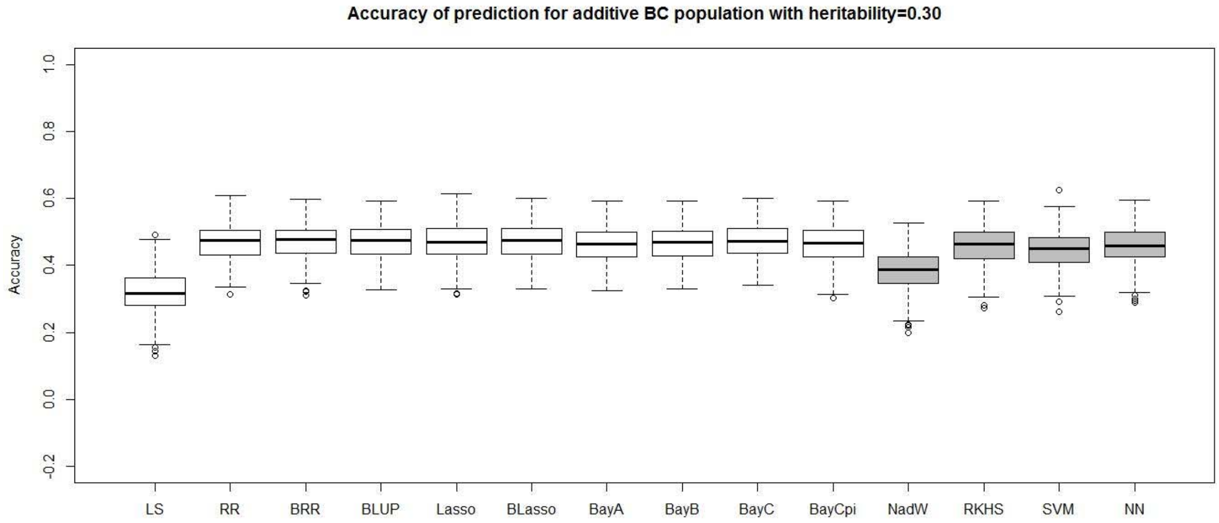

Figure 5. The boxplots of accuracy of prediction for the BC population with epistatic genetic architecture and heritability of 0.30. The first 10 boxplots correspond to the parametric methods, and the last 4 (gray) boxplots correspond to the nonparametric methods.

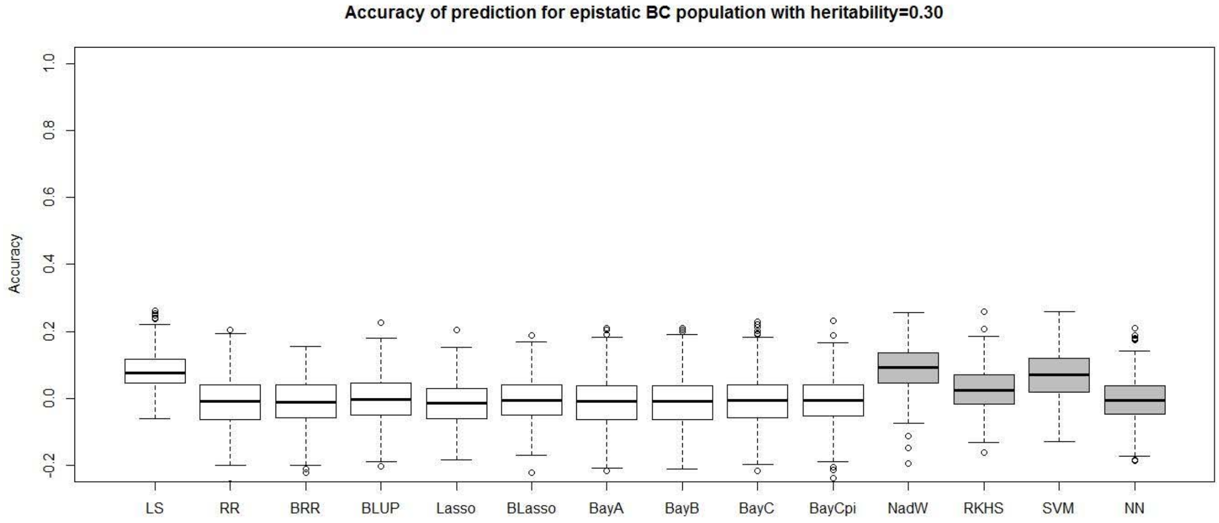

Figure 6. The boxplots of mean squared error for the BC population with additive genetic architecture and heritability of 0.70. The first 10 boxplots correspond to the parametric methods, and the last 4 (gray) boxplots correspond to the nonparametric methods.

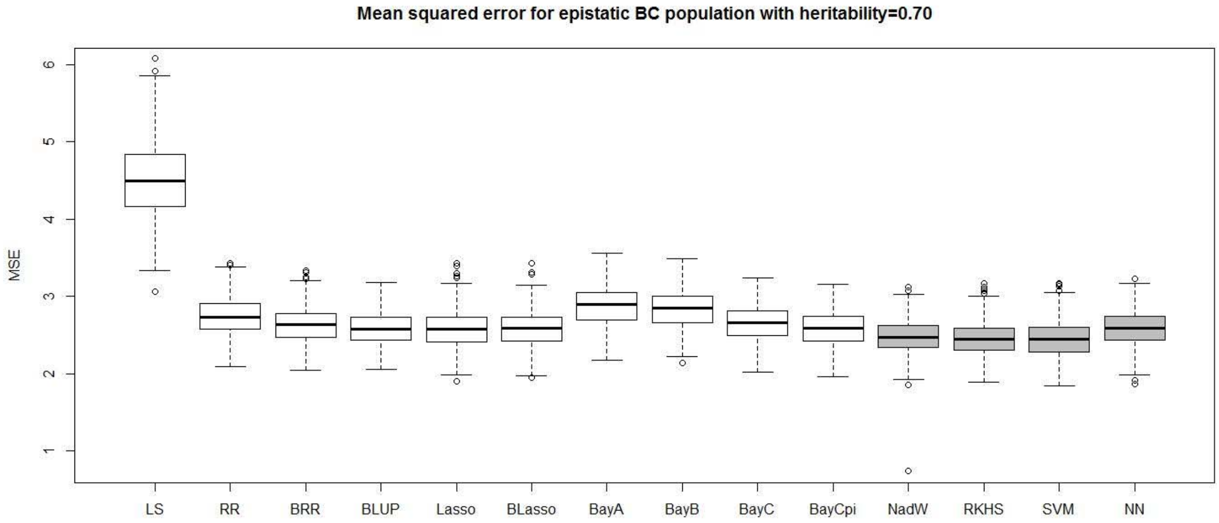

Figure 7. The boxplots of mean squared error for the BC population with epistatic genetic architecture and heritability of 0.70. The first 10 boxplots correspond to the parametric methods, and the last 4 (gray) boxplots correspond to the nonparametric methods.

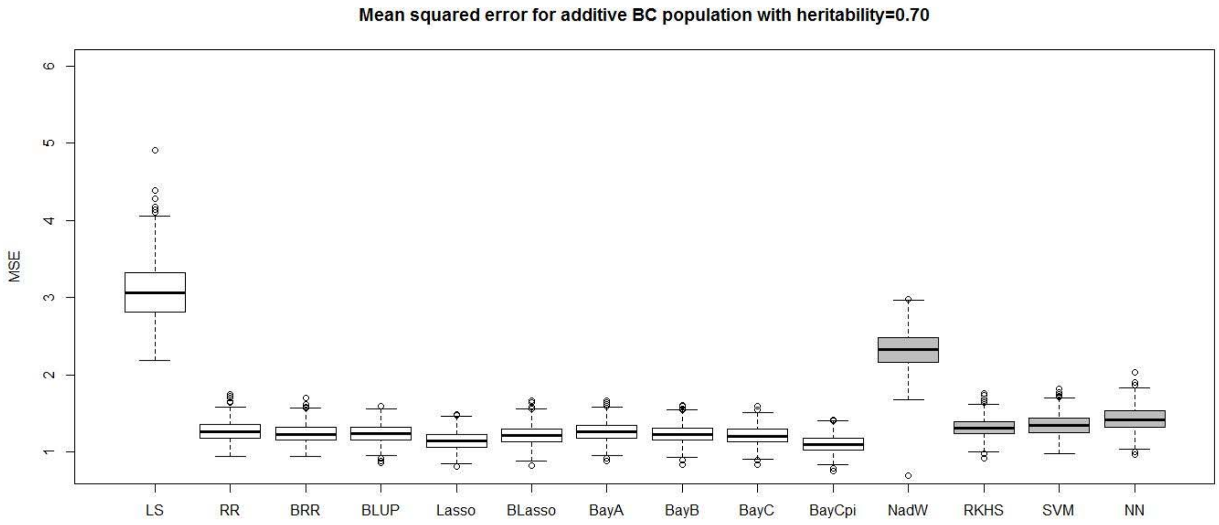

Figure 8. The boxplots of mean squared error for the BC population with additive genetic architecture and heritability of 0.30. The first 10 boxplots correspond to the parametric methods, and the last 4 (gray) boxplots correspond to the nonparametric methods.

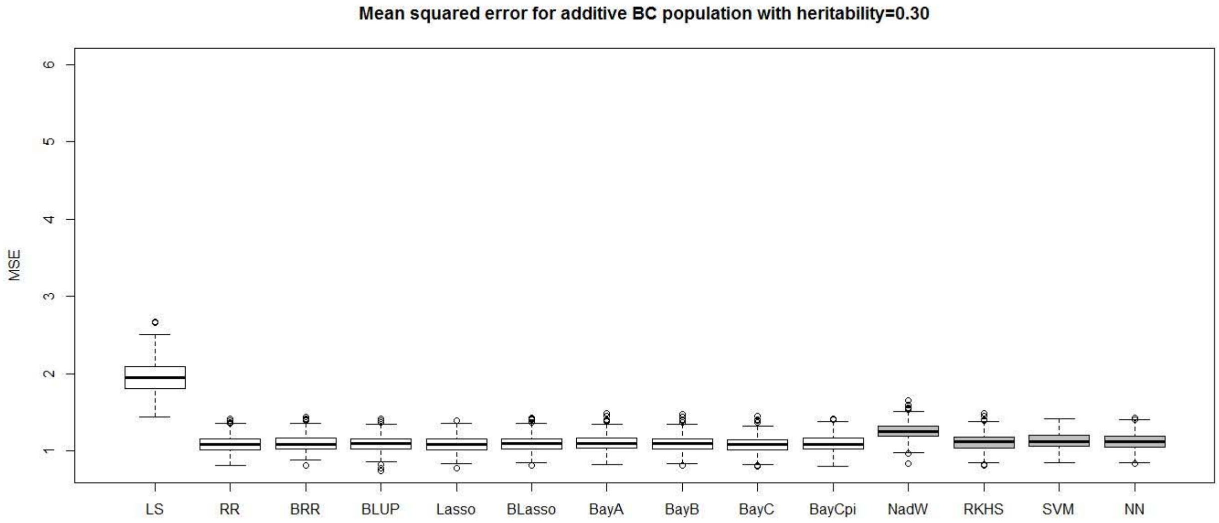

Figure 9. The boxplots of mean squared error for the BC population with epistatic genetic architecture and heritability of 0.30. The first 10 boxplots correspond to the parametric methods, and the last 4 (gray) boxplots correspond to the nonparametric methods.

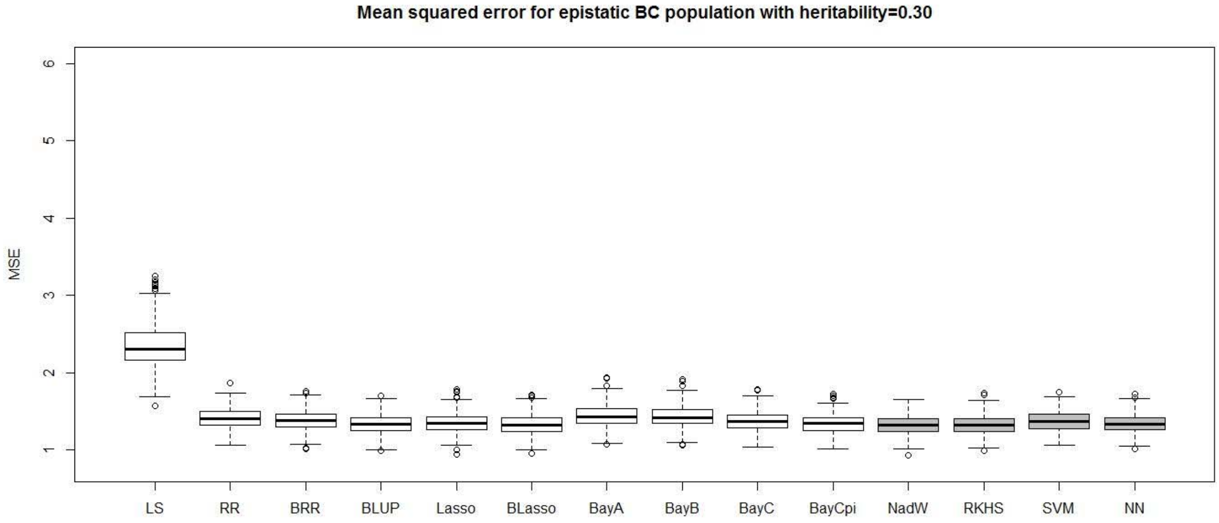

Figure 10. Plots of the parametric to nonparametric accuracy and MSE ratios. The left side of the plots shows the additive cases, and the right side of the plots shows the epistatic cases.

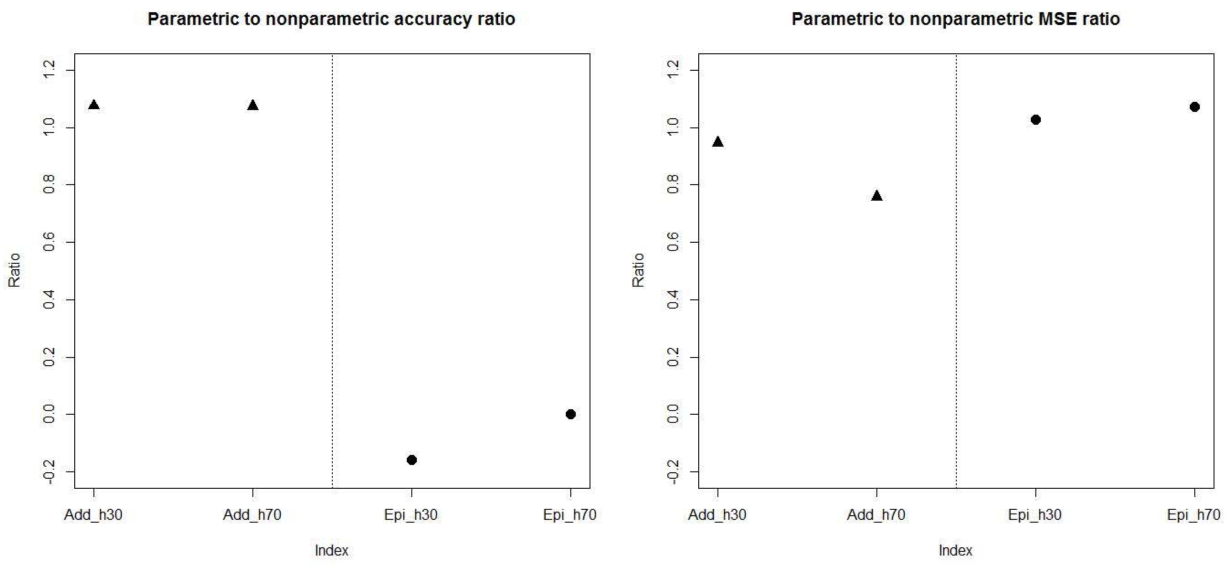

Supplement: Supporting Information [file supp_g3.114.010298_010298SI.pdf]
